# Terpenoids from Marine Sources: A Promising Avenue for New Antimicrobial Drugs

**DOI:** 10.3390/md22080347

**Published:** 2024-07-28

**Authors:** Xiao Liu, Jianzeng Xin, Yupei Sun, Feng Zhao, Changshan Niu, Sheng Liu

**Affiliations:** 1 School of Pharmacy, Yantai University, Yantai 264005, China; lx305221148@163.com (X.L.); syp6935@163.com (Y.S.); 2 Yantai Key Laboratory of Characteristic Agricultural Bioresource Conservation & Germplasm Innovative Utilization, School of life sciences, Yantai University, Yantai 264005, China; jianzeng77@sina.com; 3 College of Pharmacy, University of Utah, Salt Lake City, UT 84108, USA; niucs88@gmail.com

**Keywords:** terpenoids, antibiotic, marine, antibacterial, antifungal, minimum inhibitory concentration

## Abstract

Currently, there is an urgent need for new antibacterial and antifungal agents to combat the growing challenge of antibiotic resistance. As the largest ecosystem on Earth, the marine ecosystem includes a vast array of microorganisms (primarily bacteria and fungi), plants, invertebrates, and vertebrates, making it a rich source of various antimicrobial compounds. Notably, terpenoids, known for their complex structures and diverse bioactivities, are a significant and promising group of compounds in the battle against bacterial and fungal infections. In the past five years, numerous antimicrobial terpenoids have been identified from marine organisms such as bacteria, fungi, algae, corals, sea cucumbers, and sponges. This review article provides a detailed overview of 141 terpenoids with antibacterial and/or antifungal properties derived from marine organisms between 2019 and 2024. Terpenoids, a diverse group of natural organic compounds derived from isoprene units, are systematically categorized based on their carbon skeleton structures. Comprehensive information is provided about their names, structures, biological sources, and the extent of their antibacterial and/or antifungal effectiveness. This review aims to facilitate the rapid identification and development of prospective antimicrobials in the pharmaceutical sector.

## 1. Introduction

Antibiotics represent one of the most effective drugs for developing infections in humans and animals. Their extensive use is due to their broad spectrum of activity, which includes inhibiting the biosynthesis of the bacterial cell wall, disrupting the integrity of the cell membrane, suppressing the synthesis of nucleic acids and proteins, and interfering with metabolic processes [1].

Unfortunately, the advent of antibiotics has been accompanied by the escalating problem of antimicrobial drug resistance. In addition to inherent resistance, bacteria can acquire resistance to specific antimicrobial agents by transferring genetic material that confers resistance. To date, some of the most commonly observed strategies of bacterial resistance include modification of antibiotic target sites, increased cell wall permeability to antibiotics, active expulsion of antibiotics from the cell (known as efflux systems), and enzymatic inactivation [2]. Antibiotic resistance is a significant global public health concern, with an estimated 1.27 million deaths worldwide attributed to it [3]. It is projected that by 2050, the global death toll due to antibiotic resistance could reach 10 million per year, up from the current estimate of 700,000 deaths per year [4]. The widespread use of antibiotics in clinical and community settings and livestock and crop production is considered one of the main drivers of antimicrobial resistance [5,6,7]. The widespread use of antibiotics in clinical and community settings and livestock and crop production is considered one of the main drivers of antimicrobial resistance. Thus, it is necessary to improve the appropriate use of antibiotics and reduce unnecessary use. The World Health Organization has identified the ESKAPE pathogens—vancomycin-resistant *Enterococcus faecalis* (VRE), methicillin-resistant *Staphylococcus aureus* (MRSA), *Klebsiella pneumoniae* (*K. pneumoniae*), *Acinetobacter baumannii* (*A. baumannii*), *Pseudomonas aeruginosa* (*P. aeruginosa*), and vancomycin-resistant Enterobacter—as those with increasing multidrug resistance. Additionally, in epidemiology, *Escherichia coli* (*E. coli*), penicillin-resistant *Streptococcus pneumoniae* (PRSP), and extensively drug-resistant (XDR) *Mycobacterium tuberculosis* (*M. tuberculosis*) are well-known and significant multidrug-resistant bacteria [8,9,10]. The urgent need for new types of antibiotics to combat these pathogens highlights the importance of discovering and developing new antibacterial products for human, animal, agricultural, food, and environmental health [11].

It is well recognized that the marine ecosystem, the largest and most significant ecosystem on Earth, boasts immense biodiversity, including organisms ranging from nanoscale microorganisms to whales [12]. The marine environment offers a higher likelihood of discovering new antibacterial drug leads than terrestrial environments, making it a promising source for developing new antibiotics. Various marine organisms, such as bacteria, fungi, algae, corals, sea cucumbers, and sponges, have been explored for isolating antibacterial and antifungal bioactive compounds [13].

**Figure 1:**
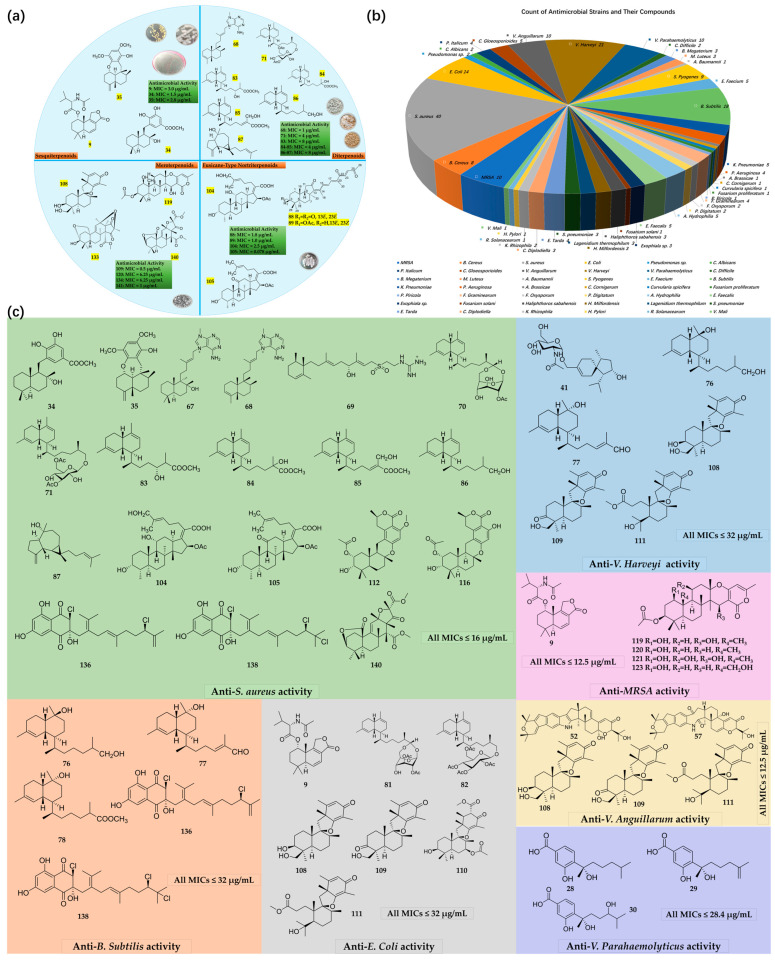
Illustration of statistical data analysis: (**a**) representative terpenoid compounds with antimicrobial activity; (**b**) count of antimicrobial strains and their corresponding compounds. (**c**) Terpenoid compounds with selective antimicrobial activity against targeted bacterial strains.

**Figure 2:**
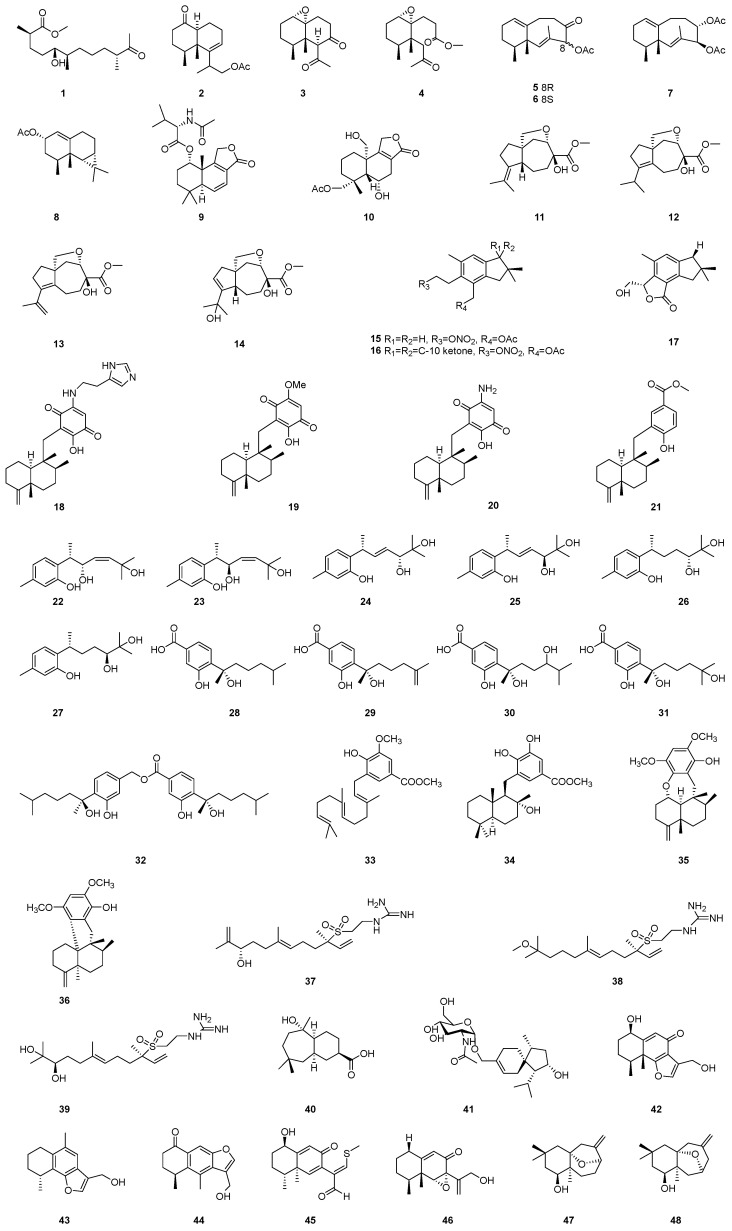
Chemical structures of sesquiterpenoids (**1**–**48**).

**Figure 3:**
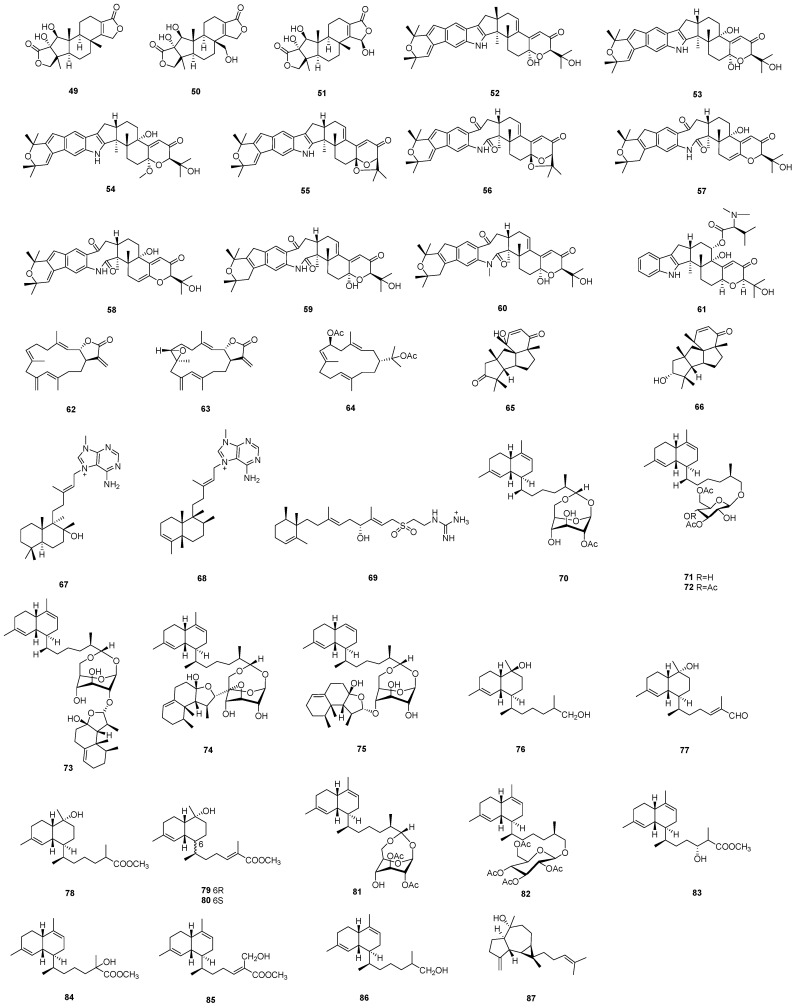
Chemical structures of diterpenoids (**49**–**87**).

**Figure 4:**
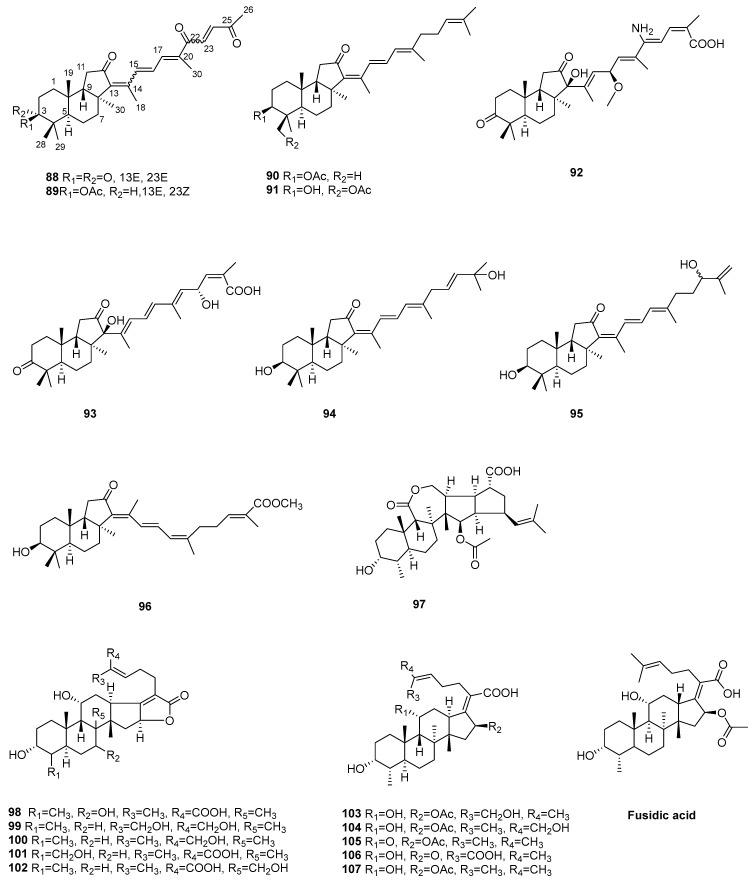
Chemical structures of triterpenoids (**88**–**107**).

**Figure 5:**
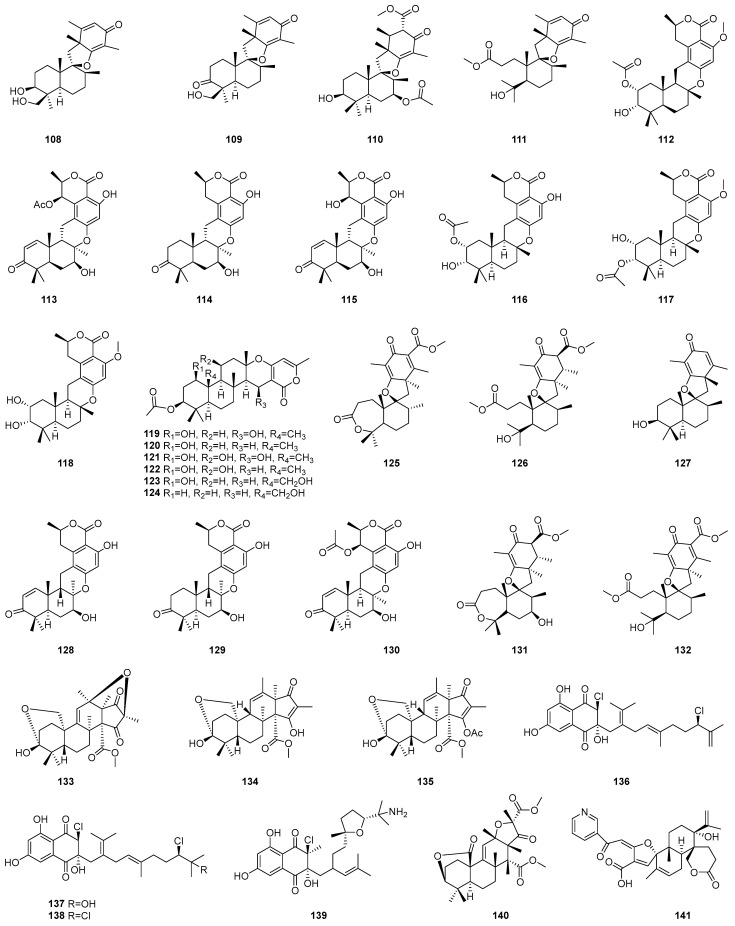
Chemical structures of meroterpenoids (**108**–**141**).

Terpenoids, significant both as natural products from terrestrial microorganisms and as metabolites in the ocean, are key candidates in the fight against microbial infections [14]. This review article provides a comprehensive account of 143 terpenoids identified between 2019 and 2024, with antibacterial and/or antifungal activities, sourced from a diverse array of marine organisms, including bacteria, fungi, algae, corals, sea cucumbers, and sponges. It details the names, structures, biological origins, and the compounds’ effectiveness against drug-resistant pathogens (most entries include the minimum inhibitory concentration (MIC) values against test bacterial and/or fungal strains). Additionally, certain compounds’ structure–activity relationships (SARs) were analyzed based on the magnitude of antimicrobial activity. The structures of these compounds are depicted in Figures 2–5, while the remaining information is presented in Tables 1–8. Figure 1 illustrates the analysis of statistical data. This review aims to facilitate and accelerate the identification and development of potentially innovative antimicrobial compounds to advance new pharmaceutical options.

## 2. Chemical Constitution

Terpenes are a diverse group of natural products synthesized from repeating units of isoprene. This class includes various compounds, such as monoterpenes (C10), sesquiterpenes (C15), diterpenes (C20), and triterpenoids (C30). Frequent skeletal rearrangements within these structures often deviate from the typical head-to-tail arrangement of isoprene units, introducing a significant degree of diversity to the terpenoid framework [13,15]. This review encompasses 141 antimicrobial terpenoids, including 48 sesquiterpenoids, 39 diterpenoids, 20 triterpenoids, and 34 meroterpenoids.

### 2.1. Sesquiterpenoids (***1**–**48***)

Sesquiterpenes are characterized by their basic carbon skeleton, which includes 15 carbon atoms arranged in three isoprene units. This section introduces 48 sesquiterpenoid compounds, including one linear sesquiterpenoid, three nardosinane-type sesquiterpenes, three neolemnane sesquiterpenes, one aristolane-type sesquiterpenoid, two drimane sesquiterpenes, and four each of carotane-style, merosesquiterpenoids, and three illudalane-style sesquiterpenoid. Additionally, there are eleven bisabolane-type sesquiterpenoids and eight sesquiterpene-derived compounds, such as sesquiterpene hydroquinones and sesquiterpene glycosides, and seven unclassified sesquiterpenoids.

The chemical structures of sesquiterpenoids **1**–**48** are depicted in Figure 2, while the remaining information, including names and marine sources, is presented in Table 1.

#### 2.1.1. Linear Sesquiterpenoid

Chermesiterpenoid D (**1**), a new linear sesquiterpenoid, was isolated and identified from the fungus *Penicillium rubens* AS-130, which originates from the Magellan Seamount. The elucidation of its structure was achieved through nuclear magnetic resonance (NMR) and mass spectroscopic (MS) data analysis. The determination of its absolute configuration was accomplished by employing a synergistic approach of quantum mechanics (QM)-NMR and time-dependent density functional theory (TDDFT) computational methods [16].

#### 2.1.2. Nardosinane-Type Sesquiterpenes

Three undescribed nardosinane-type sesquiterpenes, including 12-O-acetyl-nardosinan-6-en-1-one (**2**), 6β-acetyl-1(10)-α-13-nornardosin-7-one (**3**), and 6α-acetyl-1(10)-α-13-nornardosin-7-one (**4**), were isolated from the alcyonacean soft coral *Rhytisma fulvum fulvum*. Their chemical structures were elucidated based on 1D, 2D NMR, and MS spectral data [17].

#### 2.1.3. Neolemnane Sesquiterpenes and Aristolane-Type Sesquiterpenoids

Three novel neolemnane sesquiterpenes, designated as Lineolemnenes E, F, and G (**5**–**7**), along with a new aristolane-type sesquiterpenoid, 2-acetoxy-aristolane (**8**), have been characterized. Their structural elucidation was achieved through comprehensive spectroscopic analyses coupled with the comparison of experimental and calculated electronic circular dichroism (ECD) data [18].

#### 2.1.4. Drimane Sesquiterpenes

A marine-derived *Penicillium* sp. ZZ1283 yielded a novel drimane sesquiterpene lactone, purpuride D (**9**). The structure of purpuride D was elucidated through a multifaceted approach that included high-resolution electrospray ionization mass spectrometry (HRESIMS), NMR spectroscopic analyses, single-crystal X-ray diffraction, and ECD calculations [19]. Additionally, another drimane sesquiterpenoid, astellolide Q (**10**), was isolated from the culture of the marine fungus *Penicillium* sp. N-5. Its structure was also determined by a combination of spectroscopic methods, including MS, NMR, ECD, and X-ray diffraction [20].

#### 2.1.5. Carotane-Style Sesquiterpenoids

Byssocarotins A−D (**11**–**14**), four new carotane-style sesquiterpenoids, were obtained from a macroalga-associated strain (RR-dl-2-13) of the fungus *Byssochlamys spectabilis*. These isolates were identified through an integrated application of various spectroscopic techniques, encompassing MS, NMR, ECD, and X-ray diffraction [21].

#### 2.1.6. Illudalane Sesquiterpenoids

From an Antarctic deep-water octocoral, four bioactive compounds were successfully isolated, comprising three illudalane sesquiterpenoids: Alcyopterosin T (**15**), Alcyopterosin U (**16**), and Alcyopterosin V (**17**). The structural characterization of these novel entities was accomplished by employing a thorough suite of 1D and 2D NMR analytical techniques [22].

#### 2.1.7. Merosesquiterpenoids

Four merosesquiterpenoids, including a new sesquiterpenoid aminoquinone known as nakijiquinone V (**18**), were identified from the Indonesian marine sponge *Dactylospongia elegans*. Additionally, illimaquinone (**19**), smenospongine (**20**), and dyctioceratine C (**21**) were also found. The structure of compound **18** was elucidated by 1D and 2D NMR as well as by liquid chromatography HRESIMS data analysis [23].

#### 2.1.8. Bisabolane-Type Phenolic Sesquiterpenoids

From the South China Sea marine sponge *Plakortis simplex*, a collection of six novel bisabolane-type phenolic sesquiterpenoids was successfully isolated, including plakordiols A to D (**22**–**25**), (7R, 10R)-hydroxycurcudiol (**26**), and (7R, 10S)-hydroxycurcudiol (**27**). These compounds were extracted from the methanolic extract of *Plakortis simplex* through a series of reversed-phase chromatography and RP-HPLC separation techniques. The elucidation of their structures was facilitated by MS and NMR spectroscopy. Furthermore, compounds **22**–**27**’s stereochemical configurations were determined by combining coupling constant analysis, NOESY correlations, and applying the modified Mosher’s method [24].

Bisabolene derivatives, designated as compounds **28**–**31**, and a bisabolene dimer (**32**), were successfully isolated and characterized from *Aspergillus versicolor* AS-212, an Endozoic Fungus associated with Deep-Sea Coral of Magellan Seamounts. The chemical structures were ascertained through a comprehensive analysis of spectroscopic data, X-ray crystallography, specific rotation, ECD calculations, and spectral comparison [25].

#### 2.1.9. Sesquiterpene-Derived Compounds

Four new sesquiterpene hydroquinones, named xishaeleganins A–D (compounds **33**–**36**), have been successfully isolated from the Xisha marine sponge *Dactylospongia elegans* (family Thorectida). Their structures were determined through a thorough examination using spectroscopic methods, ECD computations, and corroboration with spectral data documented in existing literature [26].

Three novel 2-guanidinoethanesulfonyl sesquiterpene analogs of (−)-agelasidine A, designated agelasidines G–I numbered compounds **37**–**39**, were isolated from a marine sponge *Agelas nakamurai*, which was collected in Orchid Island. The absolute configurations for compounds **37**–**39** were ascertained by applying computational NMR data, the statistical DP4+ protocol, and correlating their experimental optical rotations with values predicted by B3LYP calculations [27].

Malfilanol C (**40**), a novel sesquiterpenoid, was first identified from the *Aspergillus genus* through its successful isolation from the rice solid-state fermentation products of the deep-sea-derived fungus *Aspergillus puniceus* A2. Its structure was elucidated based on comprehensive spectroscopic analysis, including HRESIMS and NMR, and comparing experimental and calculated ECD spectra to determine the absolute configuration [28].

A sesquiterpene glycoside, trichoacorside A (**41**), was isolated and identified from the culture extract of Trichoderma *longibrachiatum* EN-586, an endophytic fungus, obtained from the marine red alga Laurencia obtuse. The structures were deciphered from NMR and MS data, with absolute configurations confirmed through X-ray crystallography, derivatization, and DP4+ analysis [29].

#### 2.1.10. Others Sesquiterpenoids

From the cultured mangrove-derived fungus *Penicillium* sp. HDN13-494, five novel sesquiterpenoids have been isolated: citreobenzofurans D to F (**42**–**44**) and phomenones A to B (**45**–**46**). Their structures were identified through comprehensive spectroscopic analysis, HRESIMS, and ECD calculations. Additionally, the absolute structure of compound **43** was confirmed by single-crystal X-ray diffraction [30].

Two novel sesquiterpenoids, identified as O8-ophiocomane (**47**) and O7-ophiocomane (**48**), have been successfully isolated from the brittle star *Ophiocoma dentata*, locally sourced from the Red Sea coast of Egypt. The structures were determined using 1D and 2D NMR, FT-IR, and MS [31].

### 2.2. Diterpenoids (***49**–**87***)

Diterpenoids are a class of naturally occurring secondary metabolites with complex and diverse structures and biological activities, composed of 20 carbon atoms with extensive skeletal rearrangements [32]. This section presents 39 diterpenoid compounds, encompassing three 5,5,6,6,5-pentacyclic spongian diterpenes, nine indole diterpenes, one indole diterpene amino acid conjugate, three membrane diterpenes, two cyclopiane diterpenes, three diterpene alkaloids, six bicyclic diterpene glycosides, seven biflorane-type diterpenoids, and four decalin-type bicyclic diterpenes. The chemical structures of diterpenoids **49**–**87** are depicted in Figure 3, while the remaining information, including names and marine sources, is presented in Table 2.

#### 2.2.1. 5,5,6,6,5-Pentacyclic Spongian Diterpenes

From a Red Sea sponge specimen identified as *Spongia* sp., three novel 5,5,6,6,5-pentacyclic spongian diterpenes, designated Spongenolactones A–C and numbered **49**–**51**, have been isolated. Their structures were determined through comprehensive spectroscopic analysis, and the absolute configurations were ascertained by comparing experimental circular dichroism (CD) spectra with calculated ECD spectra [33].

#### 2.2.2. Indole Diterpenes

Nine novel indole diterpenes, designated as Janthinellumine A through I and numbered **52**–**60**, were isolated from the co-culture of two marine-derived fungi, *Penicillium janthinellium* and *Paecilomyces formosus*. The chemical structures and absolute configurations were determined using extensive spectroscopic data and computational ECD and vibrational circular dichroism (VCD) methods [34].

A rare indole diterpene amino acid conjugate, noonidole A (**61**), was derived from the marine fungus *Aspergillus* sp. CMB-M0339 (identified as *Aspergillus noonimiae*). Its structure was determined through MS spectroscopy and X-ray crystallography [35].

#### 2.2.3. Cembrane Diterpenes

Three novel cembrane diterpenes, Nephthecrassocolides A and B (**62**–**63**), along with 6-acetoxynephthenol acetate, have been isolated from a population of the marine organism *Nephthea* sp. Their structures were determined using spectroscopic methods, including MS, NMR, and nuclear Overhauser effect spectroscopy (NOESY) [36].

#### 2.2.4. Cyclopiane Diterpenes

Two novel cyclopiane diterpenes, 4-Hydroxyleptosphin C (**65**) and 13-epi-conidiogenone F (**66**), have been isolated from the marine sediment-derived fungus *Penicillium antarcticum* KMM 4670. Their absolute configurations were confirmed by the modified Mosher method and ECD spectrum calculations [37].

#### 2.2.5. Diterpene Alkaloids

Three new diterpene alkaloids, (+)-8-epiagelasine T (**67**), (+)-10-epiagelasine B (**68**), and (+)-12-hydroxyagelasidine C (**70**), have been isolated from the sponge *Agelas citrina*, which was collected along the coasts of the Yucatan Peninsula. Their structures were identified through NMR spectroscopy, HRESIMS, and literature comparison [38].

#### 2.2.6. Bicyclic Diterpene Glycosides

From the soft coral *Lemnalia bournei*, six new bicyclic diterpene glycosides—lemnaboursides E to G (**70**–**72**) and lemnadiolboursides A to C (**73**–**75**)—were meticulously isolated and characterized. Their structures were determined using spectroscopy (MS, NMR, heteronuclear single quantum coherence (HSQC), heteronuclear multiple bond correlation (HMBC), HRESIMS), ECD analysis, optical rotation, and literature data comparison [39].

#### 2.2.7. Biflorane-Type Diterpenoids

A series of novel secondary metabolites, comprising five new biflorane-type diterpenoids designated as biofloranates E through I (**76**–**80**) and two new bicyclic diterpene glycosides named lemnaboursides H through I (**81**–**82**), have been successfully isolated from the soft coral *Lemnalia bournei*, collected from the South China Sea. Their chemical structures and stereochemistry were identified using various spectroscopic techniques and TDDFT ECD calculations and were confirmed by comparing them with reported values [40].

#### 2.2.8. Decalin-Type Bicyclic Diterpenes

Four novel decalin-type bicyclic diterpenes, designated Biofloranates A to D (**83**–**86**), along with a new aromadendrane-type diterpenoid (**87**), have been successfully isolated from the soft coral *Lemnalia* sp., collected from the South China Sea. The new compounds’ structures were determined using NMR, Mosher’s method, and ECD analysis [18].

### 2.3. Triterpenoids (***88**–**107***)

Triterpenoids are a class of ubiquitous natural organic compounds in nature, consisting of six isoprene units, totaling 30 carbon atoms. This section introduces 20 triterpenoid compounds, including details on the names, sources, and structures of the nine isomalabaricane terpenoids and eleven fusicane-type nortriterpenoids. The chemical structures of triterpenoids **88**–**107** are depicted in Figure 4, while the remaining information, including names and marine sources, is presented in Table 3.

#### 2.3.1. Isomalabaricane Terpenoids

Nine novel isomalabaricane terpenoids, numbered **88**–**96**, have been successfully isolated from the sponge *Rhabdastrella globostellata* collected from Ximao Island. The structures were determined using spectroscopic methods, including MS, NMR, HMBC, and ECD, and by comparing them with known compounds’ data [41].

#### 2.3.2. Fusicane-Type Nortriterpenoids

Eleven novel fusicane-type nortriterpenoids, named Implifusidic acids A–K and numbered **97**–**107**, have been successfully isolated from the marine-derived fungus *Simplicillium* sp. SCSIO 41513. Their structures were identified through spectroscopy, and the absolute configurations were confirmed by ECD calculations, spectral comparison, and X-ray diffraction [42].

### 2.4. Meroterpenoids (***108**–**141***)

Meroterpenoids are hybrid secondary metabolites from mixed biosynthetic pathways, partially derived from terpenoid substrates. These compounds, produced widely by bacteria, algae, plants, and animals, exhibit remarkable chemical diversity by combining terpenoid frameworks with polyketides, alkaloids, phenols, and amino acids [43,44,45]. The chemical structures of meroterpenoids **108**–**141** are depicted in Figure 5, while the remaining information, including names, marine sources, and results of antibacterial activity assays, is presented in Table 4.

Four novel meroterpenoids, designated as chermesins E–H (**108**–**111**), were successfully isolated from *Penicillium chermesinum* EN-480, an endophyte derived from the marine red alga. The structures were ascertained using HRESIMS and NMR, with absolute configurations verified through NOESY, X-ray diffraction, and ECD cotton effect analysis [46].

#### 2.4.1. Drimane-Type Meroterpenoid

The first drimane-type meroterpenoid featuring a C10 polyketide unit with an 8R-configuration, named taladrimanin A (**112**), has been isolated alongside three biogenetically related compounds (**113**–**115**) from the marine-derived fungus *Talaromyces* sp. HM6-1–1 [47]. After two years, three new drimane-type meroterpenoids, designated as Taladrimanin B to D and numbered **116**–**118**, have been successfully isolated from the marine-derived fungus *Talaromyces* sp. M27416 [48]. The planar structure of **112** was identified using HRESIMS and NMR. Its relative configuration was deduced through quantum chemical NMR calculations of potential isomers and the DP4+ method. X-ray diffraction confirmed the relative and absolute configurations. Compound **116**’s structure was elucidated by HRESIMS and NMR, with configuration confirmed by quantum chemical analysis and the DP4+ method and verified by X-ray crystallography. ECD calculations established the absolute configuration of **116**, while comparative NMR and ECD analyses determined those of **117** and **118**.

#### 2.4.2. α-Pyrone Meroterpenoids

Six new α-pyrone meroterpenoids, designated as chevalones H–M and numbered **119**–**124**, have been successfully isolated from *Aspergillus hiratsukae* SCSIO 7S2001, a fungus derived from the gorgonian coral collected at Mischief Reef in the South China Sea. The structures and absolute configurations were determined by spectroscopy and X-ray diffraction [49].

#### 2.4.3. Spiromeroterpenoids

The chemical exploration of the ethyl acetate (EtOAc) extract derived from the fermentation broth of the marine fungus *Trametes* sp. ZYX-Z-16 yielded eight meroterpenoids, numbered **125**–**132**, among which two novel spiromeroterpenoids, named asnovolin H (**131**) and asnovolin I (**132**), were identified. The structures of **131** and **132** were identified through 1D and 2D NMR, HRESIMS, and ECD spectral analysis [50].

#### 2.4.4. Andrastin-Type Meroterpenoids

In addition to compound **10**, three andrastin-type meroterpenoids, identified as Hemiacetalmeroterpenoids A to C (**133**–**135**), were isolated from the marine-derived fungus *Penicillium* sp. N-5. Its structure was also determined by a combination of spectroscopic methods, including MS, NMR, ECD, and X-ray diffraction [20].

#### 2.4.5. Chlorinated Meroterpenoids

From the cultivation of the marine sediment-derived bacterium strain *Streptomyces* sp. CNH-189, four novel chlorinated meroterpenoids, identified as merochlorins G through J (**136**–**139**), have been successfully isolated. The planar structures of compounds **137**−**140** were deduced from MS, ultraviolet-visible spectroscopy, and NMR data. Their relative configurations were inferred from nuclear Overhauser effect (NOE) data, and absolute configurations were confirmed by comparing ECD spectra with known models and DP4 calculations [51].

#### 2.4.6. 3,5-Dimethylorsellinic Acid-Based Meroterpenoid

A chemical investigation of the extracts from the fungus *Aspergillus* sp. CSYZ-1 has led to the identification of aspergillactone (**140**), a novel 3,5-dimethylorsellinic acid-based meroterpenoid. NMR and mass spectrometry confirmed the structure and relative configuration of **140**. Its absolute configuration was ascertained through TDDFT calculations and comparison with experimental ECD spectra [52].

#### 2.4.7. Meroterpenoid-Type Alkaloid

Oxalicine C (**141**), a novel meroterpenoid-type alkaloid, has been isolated from the endophytic fungus *Penicillium chrysogenum* XNM-12, derived from marine algae. The planar structure of compound **141** was elucidated through spectroscopic analyses comprising ultraviolet–visible spectroscopy, 1D and 2D NMR, and HRESIMS. Its stereochemical configuration was determined by comparing experimental and calculated ECD spectra [53].

## 3. Antibacterial and/or Antifungal Activity

### 3.1. Sesquiterpenoids

This section provides a detailed account of the antimicrobial activities of 48 sesquiterpenoid compounds, with further details presented in Table 5.

Chermesiterpenoid D (**1**) demonstrated weak antibacterial activity, with MIC values for *MRSA* being 64 µg/mL [16]. Compounds **2**–**4** have exhibited against several Gram-positive (*Bacillus cereus* (*B. cereus*), *Staphylococcus aureus* (*S. aureus*)) and Gram-negative (*E. coli*, *K. pneumoniae,* and *Pseudomonas* sp.) bacteria. However, they have not shown inhibition against fungi *Aspergillus niger* (*A. niger*), *Fusarium oxysporum* (*F. oxysporum*), and *C. albicans* (*C. albicans*) [17].

Unfortunately, three new precious neolemnane sesquiterpene lineolemnenes, E, F, G (**5**–**7**), and a new aristolane-type sesquiterpenoid, 2-acetoxy-aristolane (**8**), have tested antibacterial activities against *S. aureus* and *B. cereus* with relatively high MIC values [18].

Compound **9**, a drimane sesquiterpenoid, exhibited significant antibacterial activity with the *MRSA*, *E. coli,* and *C. albicans* with MIC values of 4, 3, and 8 µg/mL, respectively [19]. Another drimane sesquiterpenoid astellolide Q (**10**) showed remarkable antifungal activities against *Penicillium italicum* (*P. italicum*) and *C. gloeosporioides* with MIC values both being of 25 µg/mL [20].

Byssocarotins A–D (**11**–**14**) displayed antagonism against the marine-derived bacteria *Vibrio parahaemolyticus* (*V. parahaemolyticus*) and *V. harveyi* with MIC values ranging from 13 to 50 µg/mL [21]. Alcyopterosin T (**15**), Alcyopterosin U (**16**), and Alcyopterosin V (**17**) were inactive against the ESKAPE panel of bacterial pathogens. However, compound **17** demonstrated significant efficacy against *Clostridium difficile* (*C. difficile*), an intestinal bacterium that is notoriously challenging to treat [22].

Compounds **18**–**21** exhibited moderate to low antibacterial activity against Bacillus megaterium (*B. megaterium*) DSM32, with MIC values of 32 µg/mL for each. Furthermore, compounds **19** and **20** inhibited Micrococcus luteus (*M. luteus*) ATCC 4698 with MIC values of 32 µg/mL [23].

Plakordiols A to D (**22**–**25**), (7R, 10R)-hydroxycurcudiol (**26**), and (7R, 10S)-hydroxycurcudiol (**27**). None of these compounds, ranging from **22** to **27**, exhibited inhibitory effects on a panel of five bacterial strains, including *S. aureus* ATCC 25923, *MRSA* ATCC 43300, *A. baumannii* ATCC 19606, *P. aeruginosa* (clinical), and *VRE* CD27. However, compounds **27** and **28** demonstrated weak antibacterial activity against *A. baumannii* ATCC 19606 in disc diffusion tests, producing inhibition zone diameters of 5 mm each. Despite this, these compounds failed to exhibit significant activity against *A. baumannii* ATCC 19606 in MIC bioassays, with MIC values exceeding 64 µg/mL [24].

Compounds **28**–**32** primarily displayed antimicrobial properties against *V. harveyi* and *V. parahaemolyticus*, with MIC values varying between 15.0 and 121.2 µg/mL. Notably, antimicrobial assays revealed that compound **29** exhibited superior efficacy to compounds **30** and **31** against the Vibrio species and *C. gloeosporioides*. These findings suggest hydroxylation at the C-10 or C-11 position may attenuate the antimicrobial activity against these microbial strains [25].

Compound **34** exhibited notable antibacterial potency, demonstrating significant inhibitory effects against *S. aureus* USA300 LAC, Streptococcus pyogenes (*S. pyogenes*) ATCC 12344, and Enterococcus faecium (*E. faecium*) Efm-HS0649. MICs for compound **34** were determined to be 1.5, 1.5, and 3.0 µg/mL for each bacterium, respectively. These MIC values are in the same range as those observed for the positive control, vancomycin, which tested a MIC of 1.0 µg/mL [26].

Remarkably, compound **37** displayed measurable antimicrobial activities when administered 25 mg per disk. This compound exhibited inhibitory effects against a panel of bacterial strains, including *Bacillus subtilis* (*B. subtilis*), *E. coli*, *K. pneumoniae*, *Salmonella typhimurium* (*S. typhimurium*), and *S. aureus*, with each strain demonstrating an inhibition zone of 3.0 mm in diameter [27]. Malfilanol C (**40**) exhibited weak antibacterial activity against *S. aureus* ATCC 29213 [28]. Sesquiterpene glycoside, trichoacorside A (**41**), demonstrated moderate activity against *MRSA* and *Vibrio harveyi* (*V. harveyi*), an aquatic pathogenic bacterium, with MIC values being 4 µg/mL, the tested plant–pathogenic fungi, including *Alternaria brassicae*, *Calonectria cornigerum*, *Colletotrichum gloeosporioides* with MIC values ranging from 8 to 64 µg/mL [29].

The majority of compounds **42**–**46**, specifically **42**–**45,** demonstrated high MIC values, indicating weak antibacterial potential. In contrast, compound **46** displayed a more pronounced effect, exhibiting moderate antibacterial activity against *B. subtilis* with an MIC value of 6.25 µg/mL [30]. Both compounds **47** and **48** have shown antibacterial efficacy against *P. aeruginosa* and *Enterococcus faecalis* (*E. faecalis*), with their antibacterial activities quantified in absolute activity units (AUs) [31].

### 3.2. Diterpenoids

This part elaborates on the antimicrobial effects of 39 diterpenoid substances (compounds **49**–**87**), with additional information found in Table 6.

Three novel 5,5,6,6,5-pentacyclic spongian diterpenes, numbered **49**–**51**, have been isolated. Subsequent in vitro assays were conducted to evaluate their growth inhibitory effects on *S. aureus*. Notably, spongenolactone A (**49**) demonstrated significant inhibitory activity, achieving 46%, 47%, and 93% growth inhibition at concentrations of 50, 100, and 200 µM, respectively. In contrast, spongenolactone B (**50**) showed comparatively lower inhibitory effects, with 24%, 42%, and 40% growth inhibition observed at the same concentration gradients [33].

Compounds **52**–**60** have exhibited various biological activities, including anti-influenza A virus, protein tyrosine phosphatase inhibitory effects, and anti-Vibrio properties. In particular, their potential to resist *Vibrio* species has attracted significant interest. Notably, compounds **52** and **59** have demonstrated weak anti-Vibrio activity against *Vibrio anguillarum* (*V. anguillarum*), with MICs of 12.5 and 25.0 µg/mL, respectively [34].

Noonidole A (**61**) exhibited moderate antifungal activity. Regrettably, it did not demonstrate antibacterial activity against bacteria, including *E. coli* ATCC 11775, *S. aureus* ATCC 25923, and *B. subtilis* ATCC 6633 [35].

Compound **62** displayed significant antifungal activity, with a MIC value of 12.5 µg/mL against the hyphal growth inhibition of *Lentinula thermophilum*. SAR analysis revealed that the differential antifungal potency between compounds **62** and **63** may be ascribed to the presence of an epoxide ring in compound **63**. Furthermore, the trisubstitution of methyl groups in the β-configuration within compound **63** could introduce steric hindrance compared to compound **62**, potentially impacting its antifungal efficacy [36].

Compound **65** demonstrated a concentration-dependent inhibitory effect on the growth of *S. aureus*, with inhibition rates of 15.3% and 29.3% at concentrations of 12.5 µM and 100 µM, respectively. Additionally, compound **65** effectively reduced biofilm formation by *S. aureus*, with prevention rates of 15.9% and 34.5% at the same concentrations. Conversely, compound **66** showed a weaker effect on *S. aureus* growth, with inhibition of 19.1% at 100 µM and no significant impact at 12.5 µM. However, it notably inhibited biofilm formation, with prevention rates ranging from 37.9% at 12.5 µM to 52.6% at 100 µM. The half-maximal inhibitory concentration (IC_50_) for the inhibition of *S. aureus* biofilm formation by compound **66** was determined to be 76.1 µM [37]

(+)-8-epiagelasine T (**67**), (+)-10-epiagelasine B (**68**), and (+)-12-hydroxyagelasidine C (**70**) did not exhibit activity against the Gram-negative pathogens *A. baumannii* ATCC 17978, *K. pneumoniae* ATCC 700603, and *P. aeruginosa* ATCC 27823. However, these compounds did demonstrate antibacterial activity against a range of Gram-positive pathogens. This group included *S. aureus* ATCC 29213, *S. aureus* USA300LAC, *Streptococcus pneumoniae* (*S. pneumoniae*) ATCC 49619, *S. pneumoniae* 549 CHUAC, *E. faecalis* ATCC 29212, *E. faecalis* 256 CHUAC and *E. faecium* 214 CHUAC [38].

Lemnaboursides E-G (**70**–**72**) and lemnadiolboursides A–C (**73**–**75**) demonstrated discernible antibacterial activity, targeting both *S. aureus* and *B. subtilis* with MIC values ranging from 4 to 16 µg/mL. A comprehensive assessment integrating antimicrobial assays with detailed structural analyses has indicated a potential correlation between the steric hindrance of the glycosides and their antimicrobial efficacy, particularly with respect to the lemnaboursides [39].

The antibacterial activities of compounds **76**–**82** were systematically evaluated against a panel of five pathogenic bacteria, including *S. aureus*, *B. subtilis*, *V. harveyi*, *S. pneumoniae*, and *E. coli*. Notably, all compounds within the series (**76**–**82**) demonstrated antibacterial efficacy against *S. aureus* and *B. subtilis*, with MICs varying from 4 to 64 µg/mL [40]. Compounds **83**–**87** exhibited antimicrobial properties, demonstrating antibacterial activity against *S. aureus* and *B. cereus* with MICs ranging from 4 to 16 µg/mL [18].

### 3.3. Triterpenoids

This section offers a comprehensive examination of the antimicrobial properties of 20 triterpenoid compounds, numbered **88** to **107**, with further details available in Table 7.

Nine novel isomalabaricane terpenoids, numbered **88**–**96**, were tested against *S. aureus* USA300LAC and *S. pyogenes* ATCC12344. In this series, compounds 89 and 90 showed a substantial antibacterial effect against *S. pyogenes*, with MIC values recorded at 1.8 and 1.0 µg/mL, respectively [41].

Compound **105** exhibited potent antibacterial activity against *S. aureus*, with a remarkably low MIC value of 0.078 µg/mL. A subset of the compounds, specifically **98**, **100**–**102**, **104**, and **105**, were selected for their targeted evaluation of antibacterial activity against *S. aureus*. However, compounds **97**, **99**, **103**, **106**, and **107** were excluded from the antibacterial panel due to their limited availability and the susceptibility of compound **97** to hydrolysis. Drawing on previous SAR studies, the chemical structure of fusidic acid has been established as optimal for antibacterial potency, with the C-21 carboxylic acid group being an essential moiety for activity [54]. The antibacterial findings for compounds **98**, **100**, **101**, **102**, **104**, and **105** corroborated this established conclusion. Furthermore, a comparative analysis of the structures and antibacterial profiles of compounds **104**, **105**, and fusidic acid indicated that the oxidation of the C-11 position to a carbonyl group did not significantly impact antibacterial efficacy. In contrast, the oxidation of the hydrophobic side chain at the C-20 position was associated with decreased antibacterial activity [42].

### 3.4. Meroterpenoids

This section details the antimicrobial properties of meroterpenoid compounds **108**–**141**, with details in Table 8.

Chermesins E–H (**108**–**111**) were subjected to a battery of assays to evaluate their antibacterial and antifungal activities against a spectrum of human and aquatic bacteria, including *Aeromonas hydrophilia* (*A. hydrophilia*), *E. coli*, *Edwardsiella tarda* (*E. tarda*), *V. anguillarum,* and *V. harveyi*, as well as against plant–pathogenic fungi, such as *Coniothyrium diplodiella* (*C. diplodiella*), and *Fusarium graminearum* (*F. graminearum*). Compound **108** displayed potent antibacterial activity against *E. tarda* and *V. anguillarum*, with MICs of 0.5 µg/mL, a value comparable to or exceeding the efficacy of the positive control, chloramphenicol, which showed MICs of 0.5 and 1 µg/mL, respectively. Additionally, compound 110 exhibited robust activity against the human pathogenic bacterium *E. coli*, with an MIC of 1 µg/mL, surpassing the activity of the positive control, chloromycetin, which had an MIC of 2 µg/mL [46].

Compound **112** exhibited selective antibacterial activity targeting *S. aureus* ATCC 6538P, demonstrating a noteworthy potency with a MIC of 15.2 µg/mL. This potency, however, was lower than that of the positive control, chloramphenicol, which had an MIC of 5.0 µg/mL. Compound **112**’s antibacterial activity was less pronounced against *V. parahaemolyticus* and *E. coli* strains [47]. Moving on to compound **116**, it also showed selective antibacterial activity against *S. aureus* CICC 10384, with an MIC of 12.5 µg/mL. This activity was on par with chloramphenicol, again serving as a positive control with an MIC of 5.0 µg/mL [48].

Compounds **119**–**124** were subjected to a broth dilution assay to evaluate their antibacterial activity against a panel of bacterial strains, including *M. luteus*, *K. pneumoniae*, *MRSA*, and *Streptococcus faecalis* (*S. faecalis*). The compounds exhibited a range of antibacterial potencies, with MICs spanning from 6.25 to 100 µg/mL [49].

Compound **129** exhibited modest inhibitory effects against *S. aureus* with a MIC of 128 µg/mL. Compounds (**125**–**128** and **130**–**132**) demonstrated lackluster antibacterial properties, with MICs exceeding 128 µg/mL for both *S. aureus* and *B. subtilis*. Subsequently, the inhibitory activities of the same set of compounds were evaluated against five phytopathogenic fungi, including *F. oxysporum* f. sp. cubense, *Fusarium* spp, *Peronophythora litchii* (*P. litchii*), *C. gloeosporioides*, *Hylocereus undatus* (*H. undatus*) utilizing the broth microdilution technique. Regrettably, under the conditions tested, none of the compounds manifested definitive inhibitory effects against the tested fungi [50].

Hemiacetalmeroterpenoids A to C (**133**–**135**) exhibit more potent antimicrobial activities against phytopathogenic fungi than their effects on bacteria. Specifically, compound **135** displayed significant antimicrobial effects against *B. subtilis*, *P. italicum,* and *C. gloeosporioides* with MIC of 6.25 µg/mL for all three organisms. Furthermore, compound **135** also demonstrated antibacterial activity against *MRSA*, with an MIC of 25 µg/mL [20].

Compound **138** exhibited robust antibacterial activity against Gram-positive strains: *B. subtilis* KCTC 1021, *K. rhizophila* KCTC 1915, and *S. aureus* KCTC 1927. The MICs for these strains were 1, 2, and 2 µg/mL, respectively. In contrast, compound **139** exhibited no significant antibacterial activity against the same strains, with all MICs greater than 128 µg/mL [51].

Compound **140** has demonstrated potent antimicrobial activity against various strains of *Helicobacter pylori* (*H. pylori*), including ATCC43504, G27, Hp159, and BY583, with MICs ranging from 1 to 4 µg/mL. Additionally, aspergillactone (**140**) exhibited significant inhibitory effects against multiple strains of *S. aureus*, such as ATCC25923, USA300, BKS231, and BKS233, with MICs in the range of 2 to 16 µg/mL [52].

Compound **141** demonstrated inhibitory effects against *E. coli*, *M. luteus,* and *Ralstonia solanacearum* (*R. solanacearum*) with MICs consistent at 8 µg/mL. However, against *P. aeruginosa*, the MIC was found to be higher at 16 µg/mL. In parallel with the antibacterial assessments, the antifungal activity of **141** was also evaluated. The compound exhibited notable antifungal activity against a panel of five plant pathogenic fungi, including *Alternaria alternata* (*A. alternata*), *Botrytis cinerea* (*B. cinerea*), *F. oxysporum*, *Penicillium digitatum* (*P. digitatum*), and *Valsa mali* (*V. mali*). Notably, the most potent activity was observed against *V. mali*, with an MIC value of 16 µg/mL [53].

## 4. Conclusions

This review summarizes 141 terpenoid compounds with antibacterial and/or antifungal activities discovered from marine biological resources between 2019 and 2024. These compounds are primarily derived from sponges, red algae, soft corals, fungi, bacteria, and marine sediments. They include 48 sesquiterpenes, 39 diterpenes, 20 triterpenes, and 34 meroterpenoids.

The antibacterial activity of these compounds is relatively evenly distributed and does not show a particular preference for any specific skeletal type. Among sesquiterpenes, compounds such as **9** and **34** exhibit significant antibacterial activity against *S. aureus*, with MICs ranging from 4 to 1.5 µg/mL. Diterpenes, including compounds **68**, **71**, **83**–**87**, also show promising antibacterial activity with MICs between 1 and 8 µg/mL. Notably, the fusidane-type nortriterpenoid compound **105** has the lowest MIC at 0.078 µg/mL, demonstrating strong antibacterial activity. Among meroterpenoids, compound **108** has significant antifungal activity against animal pathogens *V. anguillarum* and *V. harveyi*, with an MIC as low as 0.5 µg/mL. Compound **123** demonstrated potent antibacterial activity against *M. lutea* and *S. faecalis*, with MIC values of 12.5 µg/mL. Compound **140** showed strong antibacterial activity against *H. pylori* strains ATCC43504, G27, and Hp159, with MIC values as low as 1 µg/mL.

Among the 141 terpenoid compounds surveyed, those with antibacterial activity against *S. aureus* were most abundant, comprising 40 unique compounds. The count was followed by 21 compounds active against *V. harveyi*, 18 against *B. subtilis*, 14 against *E. coli*, and 10 each with efficacy against *MRSA*, *V. anguillarum*, and *V. Parahaemolyticus*. There were nine compounds with activity against *S. pyogenes*, eight compounds against *B. Cereus*, and terpenoid compounds with activity against *C. Gloeosporioides*, *E. Faecium*, *K. Pneumoniae*, *A. hydrophilia*, and *E. Faecalis* were five each. There were four compounds each against *P. Italicum*, *P. Aeruginosa*, *F. Graminearum*, and *E. Tarda*, and three against *B. megaterium*, *M. Luteus*, *Exophiala* sp., *Haliphthoros sabahensis*, *H. Milfordensis*, *Lagenidium thermophilum*, *S. pneumoniae*, and *C. Diplodiella*. The number of terpenoid compounds resistant to *F.Oxysporum*, *P.Digitatum*, *K.Rhizophila,* and *C. albicans* strains was 2, respectively. Notably, compound **17** exhibited antibacterial properties against *C. difficile*. Among the surveyed terpenoid compounds, compound **41** is the sole terpenoid with demonstrated antibacterial activity against the strains A. Brassicae, *C. Cornigerum*, *Curvularia spicifera*, *Fusarium proliferatum*, and *P. Piricola*. The sole compound exhibiting antibacterial activity against the *A. baumannii* strain is compound **27**, producing inhibition zone diameters of 5.0 ± 0.6 mm. However, the compound’s MIC exceeds 64 µg/mL. Compound **62** is the only compound that exhibits resistance against the *Fusarium solani* strain. Compound **140** is the unique compound with antibacterial resistance against *H. pylori* and has demonstrated strong antibacterial activity against *H. pylori* strains, with an MIC of 2 µg/mL. Compound **141** stands out as the sole terpenoid to demonstrate resistance against *R. solanacearum*, with an MIC value of 8 µg/mL.

Furthermore, SAR analysis indicates that hydroxylation at C-10 and C-11 in compounds **29**, **30**, and **31** may reduce antibacterial activity; the β-configuration of the trimethyl substitution in compound **63** may decrease antifungal efficacy through steric hindrance. The structural analysis of compounds **73**–**75** suggests that the spatial hindrance of glycosides is related to antimicrobial efficacy.

In conclusion, marine terpenoids have demonstrated considerable potential for development in the antimicrobial domain. This discovery is not only of profound significance to the pharmaceutical industry in the quest for novel antimicrobial agents but also holds promise for its potential applications in various fields, including animal nutrition and food preservation.

## Figures and Tables

**Table 1:** Names, classes, skeletons, and marine sources of sesquiterpenoids (**1**–**48**).

No.	Names	Classes	Marine Sources	Ref.
**1**	Chermesiterpenoid D	Linear Sesquiterpenoid	Magellan Seamount-Derived fungus *Penicillium rubens* AS-130	[16]
**2**	12-O-acetyl-nardosinan-6-en-1-one	Nardosinane Sesquiterpene	Octocoral *Rhytisma fulvum fulvum*	[17]
**3**	6β-acetyl-1(10)-α-13-nornardosin-7-one			[17]
**4**	6α-acetyl-1(10)-α-13-nornardosin-7-one			[17]
**5**	Lineolemnene E	Neolemnane Sesquiterpene	Soft coral *Lemnalia* sp.	[18]
**6**	Lineolemnene F			
**7**	Lineolemnene G			
**8**	2-acetoxy-aristolane	Aristolane Sesquiterpenoid	Soft coral Lemnalia sp.	[18]
**9**	Lactone purpuride D	Drimane Sesquiterpene	The marine-derived *Penicillium* sp. ZZ1283	[19]
**10**	Astellolide Q	Drimane Sesquiterpene	The marine-derived fungus *Penicillium* sp. N-5	[20]
**11**	Byssocarotin A	Carotane Sesquiterpenoid	Macroalga-Derived Algicolous Fungus *Penicillium rubens* RR-dl-2-13	[21]
**12**	Byssocarotin B			[21]
**13**	Byssocarotin C			[21]
**14**	Byssocarotin D			[21]
**15**	Alcyopterosin T	Illudalane Sesquiterpenoid	Octocoral *Alcyonium* sp.	[22] [22]
**16**	Alcyopterosin U			[22]
**17**	Alcyopterosin V			[22]
**18**	Nakijiquinone V	Sesquiterpenoid Aminoquinone	Indonesian marine *Dactylospongia elegans* sponge	[23]
**19**	Illimaquinone	Merosesquiterpenoid	Indonesian marine *Dactylospongia elegans* sponge	[23]
**20**	Smenospongine			[23]
**21**	Dyctioceratine C			[23]
**22**	Plakordiol A	Bisabolane Phenolic Sesquiterpenoid	The marine sponge *Plakortis simplex*	[24]
**23**	Plakordiol B			[24]
**24**	Plakordiol C			[24]
**25**	Plakordiol D			[24]
**26**	(7R, 10R)-hydroxycurcudiol			[24]
**27**	(7R, 10S)-hydroxycurcudiol			[24]
**28**	Sydonic acid	Bisabolene Sesquiterpenoid	*Aspergillus versicolor* AS-212	[25]
**29**	(S)-(+)-11-dehydrosydonic acid			[25]
**30**	(−)-10-hydroxysydonic acid			[25]
**31**	hydroxysydonic acid			[25]
**32**	Peniciaculin B			[25]
**33**	Xishaeleganins A	Sesquiterpenoid Hydroquinone	Xisha Marine Sponge *Dactylospongia elegans*	[26]
**34**	Xishaeleganins B			[26]
**35**	Xishaeleganins C			[26]
**36**	Xishaeleganins D			[26]
**37**	Agelasidine G	Sesquiterpenoid Alkaloid	Sponge *Agelas nakamurai*	[27]
**38**	Agelasidine H			[27]
**39**	Agelasidine I			[27]
**40**	Malfilanol C	Sesquiterpenoid	The deep-sea-derived fungus *Aspergillus puniceus* A2	[28]
**41**	Trichoacorside A	Sesquiterpene Glycoside	Red Alga Laurencia obtuse -Derived Endophytic Fungus Trichoderma *longibrachiatum* EN-586	[29]
**42**	Citreobenzofuran D	Sesquiterpenoid	Mangrove-Derived Fungus *Penicillium* sp. HDN13-494	[30]
**43**	Citreobenzofuran E			[30]
**44**	Citreobenzofuran F			[30]
**45**	Phomenone A			[30]
**46**	Phomenone B			[30]
**47**	O8-ophiocomane	Sesquiterpenoid	Brittle star; *Ophiocoma dentata*	[31]
**48**	O7-ophiocomane			[31]

**Table 2:** Names, classes, skeletons, and marine sources of diterpenoids (**50**–**88**).

No.	Names	Classes	Marine Sources	Ref.
**49**	Spongenolactone A	5,5,6,6,5-Pentacyclic Spongian Diterpenes	Red Sea sponge *Spongia* sp.	[33]
**50**	Spongenolactone B			[33]
**51**	Spongenolactone C			[33]
**52**	Janthinellumine A	Indole Diterpene	Co-culturing the marine-derived fungi *Penicillium janthinellium* with *Paecilomyces formosus*	[34]
**53**	Janthinellumine B			[34]
**54**	Janthinellumine C			[34]
**55**	Janthinellumine D			[34]
**56**	Janthinellumine E			[34]
**57**	Janthinellumine F			[34]
**58**	Janthinellumine G			[34]
**59**	Janthinellumine H			[34]
**60**	Janthinellumine I			[34]
**61**	Noonindole A	Indole Diterpene Amino Acid	Fungus *Aspergillus noonimiae* CMB-M0339	[35]
**62**	Nephthecrassocolide A	Cembrane Diterpene	Bornean soft coral *Nephthea* sp.	[36]
**63**	Nephthecrassocolide B			[36]
**64**	6-Acetoxy Nephthenol Acetate			[36]
**65**	4-Hydroxyleptosphin C	Cyclopiane Diterpene	The marine sediment-derived fungus *Penicillium antarcticum* KMM 4670	[37]
**66**	13-Epi-Conidiogenone F			[37]
**67**	(+)-8-Epiagelasine T	Diterpene Alkaloid	*Agelas citrina* Sponge	[38]
**68**	(+)-10-Epiagelasine B			[38]
**69**	(+)-12-Hydroxyagelasidine C			[38]
**70**	Lemnabourside E	Bicyclic Diterpene Glycoside	Soft coral *Lemnalia bournei*	[39]
**71**	Lemnabourside F			[39]
**72**	Lemnabourside G			[39]
**73**	Lemnadiolbourside A			[39]
**74**	Lemnadiolbourside B			[39]
**75**	Lemnadiolbourside C			[39]
**76**	Biofloranate E	Biflorane-Type Diterpenoid	Soft coral *Lemnalia bournei*	[40]
**77**	Biofloranate F			[40]
**78**	Biofloranate G			[40]
**79**	Biofloranate H			[40]
**80**	Biofloranate I			[40]
**81**	Lemnabourside H	Bicyclic Diterpene Glycoside	Soft coral *Lemnalia bournei*	[40]
**82**	Lemnabourside I			[40]
**83**	Biofloranate A	Decalin-Type Bicyclic Diterpene	Soft coral *Lemnalia* sp.	[18]
**84**	Biofloranate B			[18]
**85**	Biofloranate C			[18]
**86**	Biofloranate D			[18]
**87**	Cneorubin K	Aromadendrane-Type Diterpenoid	Soft coral *Lemnalia* sp.	[18]

**Table 3:** Names, classes, skeletons, and marine sources of triterpenoids (**88**–**107**).

No.	Names	Classes	Marine Sources	Ref.
**88**	13-(E)-geoditin A	Isomalabaricane Terpenoid	Sponge *Rhabdastrella globostellata*	[41]
**89**	13-(E)-isogeoditin B			[41]
**90**	3-Acetylstelliferin D			[41]
**91**	29-Acetylstelliferin D			[41]
**92**	Hainanstelletin A			[41]
**93**	Hainanstelletin B			[41]
**94**	23,24-Ene-25-hydroxystelliferin D			[41]
**95**	25,26-Ene-24-hydroxystelliferin D			[41]
**96**	Hainanstelletin C			[41]
**97**	Implifusidic acid A	Fusidane-Type Nortriterpenoid	The marine-derived fungus *Simplicillium* sp. SCSIO 41513.	[42]
**98**	Implifusidic acid B			[42]
**99**	Implifusidic acid C			[42]
**100**	Implifusidic acid D			[42]
**101**	Implifusidic acid E			[42]
**102**	Implifusidic acid F			[42]
**103**	Implifusidic acid G			[42]
**104**	Implifusidic acid H			[42]
**105**	Implifusidic acid I			[42]
**106**	Implifusidic acid J			[42]
**107**	Implifusidic acid K			[42]

**Table 4:** Names, classes, skeletons, and marine sources of meroterpenoids (**108**–**141**).

No.	Names	Classes	Marine Sources	Ref.
**108**	Chermesin E	Meroterpenoid	Red alga-derived endophytic *Penicillium chermesinum* EN-480	[46]
**109**	Chermesin F			[46]
**110**	Chermesin G			[46]
**111**	Chermesin H			[46]
**112**	Taladrimanin A	Drimane-Type Meroterpenoid	Fungus *Talaromyces* sp. HM6-1–1	[47]
**116**	Taladrimanin B	Meroterpenoid	The marine-derived fungus *Talaromyces* sp. M27416	[48]
**119**	Chevalone H	α-Pyrone Meroterpenoid	Gorgonian coral-derived fungus *Aspergillus hiratsukae* SCSIO 7S2001	[49]
**120**	Chevalone I			[49]
**121**	Chevalone J			[49]
**122**	Chevalone K			[49]
**123**	Chevalone L			[49]
**124**	Chevalone M			[49]
**125**	Asnovolin C 5′6′-dehydrohydrogen	Spiromeroterpenoid	Conch snail-derived fungus *Trametes* sp. ZYX-Z-16	[50]
**126**	Asnovolin C			[50]
**127**	Chermesin A			[50]
**128**	Chrodrimanin E			[50]
**129**	Chrodrimanin H			[50]
**130**	Thailandolide B			[50]
**131**	Asnovolin H			[50]
**132**	Asnovolin I			[50]
**133**	Hemiacetalmeroterpenoid A	Andrastin-Type Meroterpenoid	The marine-derived fungus *Penicillium* sp. N-5	[20]
**134**	Hemiacetalmeroterpenoid B			[20]
**135**	Hemiacetalmeroterpenoid C			[20]
**136**	Merochlorin G	Chlorinated Meroterpenoid	Marine sediment-derived bacterium strain *Streptomyces* sp. CNH-189	[51]
**137**	Merochlorin H			[51]
**138**	Merochlorin I			[51]
**139**	Merochlorin J			[51]
**140**	Aspergillactone	Meroterpenoid	The marine fungus *Aspergillus* sp. CSYZ-1	[52]
**141**	Oxalicine C	Meroterpenoid-Type Alkaloid	The marine-algal-derived endophytic fungus *Penicillium chrysogenum* XNM-12	[53]

**Table 5:** Antibacterial and/or antifungal activities of sesquiterpenoids (**1**–**48**).

No.	Test Strains	Activity	Bioassays	Ref.
**1**	*MRSA*	Antibacterial	MIC = 64 µg/mL	[16]
**2**	*B. cereus*	Antibacterial	diameters of inhibition zone 6 ± 0.03 mm (50 µg/mL)	[17]
	*S. aureus*		diameters of inhibition zone 5 ± 0.00 mm (50 µg/mL)	
	*E. coli*		negative	
	*Pseudomonas* sp.		diameters of inhibition zone 4 ± 0.00 mm (50 µg/mL)	
**3**	*B. cereus*	Antibacterial	diameters of inhibition zone 6 ± 0.00 mm (100 µg/mL)	[17]
	*S. aureus*		diameters of inhibition zone 5 ± 0.00 mm (100 µg/mL)	
	*E. coli*		diameters of inhibition zone 4 ± 0.00 mm (100 µg/mL)	
	*Pseudomonas* sp.		negative	
**4**	*B. cereus*	Antibacterial	diameters of inhibition zone 6 ± 0.00 mm (100 µg/mL)	[17]
	*S. aureus*		diameters of inhibition zone 5 ± 0.00 mm (100 µg/mL)	
	*E. coli*		diameters of inhibition zone 4 ± 0.00 mm (100 µg/mL)	
	*Pseudomonas* sp.		negative	
**5**	*S. aureus*	Antibacterial	MIC > 128 µg/mL	[18]
	*B. cereus*			
**6**	*S. aureus*	Antibacterial	MIC > 128 µg/mL	[18]
	*B. cereus*			
**7**	*S. aureus*	Antibacterial	MIC > 128 µg/mL	[18]
	*B. cereus*			
**8**	*S. aureus*	Antibacterial	MIC > 128 µg/mL	[18]
	*B. cereus*			
**9**	*MRSA*	Antibacterial	MIC = 4 µg/mL	[19]
	*E. coli*		MIC = 3 µg/mL	
	*C. albicans*	Antifungal	MIC = 8 µg/mL	
**10**	*MRSA*	Antibacterial	MIC >50 µg/mL	[20]
	*B. cereus*			
	*P. italicum*	Antifungal	MIC = 25 µg/mL	
	*C. gloeosporioides*			
**11**	*V. anguillarum*	Antibacterial	diameters of inhibition zone 6.3 ± 0.6 mm (50 µg/disk)	[21]
	*V. harveyi*		negative	
	*V. parahaemolyticus*		diameters of inhibition zone 6.7 ± 0.6 mm (50 µg/disk)	
**12**	*V. anguillarum*	Antibacterial	diameters of inhibition zone 6.7 ± 0.6 mm (50 µg/disk)	[21]
	*V. harveyi*		negative	
	*V. parahaemolyticus*		diameters of inhibition zone 7.3 ± 0.6 mm (50 µg/disk)	
**13**	*V. anguillarum*	Antibacterial	negative	[21]
	*V. harveyi*		negative	
	*V. parahaemolyticus*		diameters of inhibition zone 7.3 ± 0.6 mm (50 µg/disk)	
**14**	*V. anguillarum*	Antibacterial	negative	[21]
	*V. harveyi*		negative	
	*V. parahaemolyticus*		diameters of inhibition zone 6.7 ± 0.6 mm (50 µg/disk)	
**15**	ESKAPE	Inactive	inactive against the ESKAPE	[22] [22]
**16**	ESKAPE	Inactive	inactive against the ESKAPE	[22]
**17**	*C. difficile*	Antibacterial	MIC 8.1 µg/mL	[22]
	ESKAPE		inactive against the ESKAPE	
**18**	*B. megaterium* DSM32	Inactive	inactive against *B. Megaterium* DSM32	[23]
	*M. luteus* ATCC 4698		inactive against *M. Luteus* ATCC 4698	
**19**	*B. megaterium* DSM32	Antibacterial	MIC = 32 µg/mL	[23]
	*M. luteus* ATCC 4698			
**20**	*B. megaterium* DSM32	Antibacterial	MIC = 32 µg/mL	[23]
	*M. luteus* ATCC 4698			
**21**	*B. megaterium* DSM32	Antibacterial	MIC = 32 µg/mL	[23]
	*M. luteus* ATCC 4698		MIC = 64 µg/mL	
**22**	*S. aureus* ATCC 25923	Antibacterial	MIC > 64 µg/mL	[24]
	*MRSA* ATCC 43300			
	*A. baumannii* ATCC19606			
	*P. aeruginosa* (clinical)			
	*VRE* CD27			
**23**	*S. aureus* ATCC 25923	Antibacterial	MIC > 64 µg/mL	[24]
	*MRSA* ATCC 43300			
	*A. baumannii* ATCC19606			
	*P. aeruginosa* (clinical)			
	*VRE* CD27			
**24**	*S. aureus* ATCC 25923	Antibacterial	MIC > 64 µg/mL	[24]
	*MRSA* ATCC 43300			
	*A. baumannii* ATCC19606			
	*P. aeruginosa* (clinical)			
	*VRE* CD27			
**25**	*S. aureus* ATCC 25923	Antibacterial	MIC > 64 µg/mL	[24]
	*MRSA* ATCC 43300			
	*A. baumannii* ATCC19606			
	*P. aeruginosa* (clinical)			
	*VRE* CD27			
**26**	*S. aureus* ATCC 25923	Antibacterial	MIC > 64 µg/mL	[24]
	*MRSA* ATCC 43300			
	*A. baumannii* ATCC19606			
	*P. aeruginosa* (clinical)			
	*VRE* CD27			
**27**	*A. baumannii* ATCC19606	Antibacterial	diameters of inhibition zone 5.0 ± 0.6 mm, but MIC > 64 µg/mL	[24]
**28**	*V. harveyi*	Antibacterial	MIC = 15.0 µg/mL	[25]
	*V. Parahaemolyticus*			
	*C. Gloeosporioides*	Antifungal	MIC = 120.3 µg/mL	
**29**	*V. harveyi*	Antibacterial	MIC = 15.2 µg/mL	[25]
	*V. Parahaemolyticus*		MIC = 121.2 µg/mL	
	*C. Gloeosporioides*	Antifungal	MIC = 121.2 µg/mL	
**30**	*V. harveyi*	Antibacterial	MIC = 28.4 µg/mL	[25]
	*V. Parahaemolyticus*		MIC = 113.5 µg/mL	
	*C. Gloeosporioides*	Antifungal	MIC > 200 µg/mL	
**31**	*V. harveyi*	Antibacterial	MIC > 200 µg/mL	[25]
	*V. Parahaemolyticus*			
	*C. Gloeosporioides*	Antifungal	MIC > 200 µg/mL	
**32**	*V. harveyi*	Antibacterial	MIC > 200 µg/mL	[25]
	*V. Parahaemolyticus*		MIC = 64 µg/mL	
	*C. Gloeosporioides*	Antifungal	MIC > 200 µg/mL	
**33**	*S. aureus* USA300 LAC	Inactive	inactive against *S. aureus* USA300 LAC	[26]
	*S. pyogenes* ATCC 12344		inactive against *S. pyogenes* ATCC 12344	
	*E. Faecium* Efm-HS0649		inactive against *E. Faecium* Efm-HS0649	
**34**	*S. aureus* USA300 LAC	Antibacterial	MIC = 1.5 µg/mL	[26]
	*S. pyogenes* ATCC 12344		MIC = 1.5 µg/mL	
	*E. Faecium* Efm-HS0649		MIC = 3.0 µg/mL	
**35**	*S. aureus* USA300 LAC	Antibacterial	MIC = 11.1 µg/mL	[26]
	*S. pyogenes* ATCC 12344		MIC = 2.8 µg/mL	
	*E. Faecium* Efm-HS0649		MIC = 5.6 µg/mL	
**36**	*S. aureus* USA300 LAC	Antibacterial	MIC > 186.0 µg/mL	[26]
	*S. pyogenes* ATCC 12344		MIC = 11.6 µg/mL	
	*E. Faecium* Efm-HS0649		MIC > 186.0 µg/mL	
**37**	*B. subtilis*	Antibacterial	diameters of inhibition zone 3.0 mm (25 mg/disk)	[27]
	*E. coli*			
	*K. Pneumoniae*			
	*S. aureus*			
**38**	*B. subtilis*	Inactive	inactive against *B. subtilis*	[27]
	*E. coli*		inactive against *E. coli*	
	*K. Pneumoniae*		inactive against *K. Pneumoniae*	
	*S. aureus*		inactive against *S. aureus*	
**40**	*S. aureus* ATCC 29213	Antibacterial	diameters of inhibition zone 8 mm (200 mg/disk)	[28]
**41**	*E. coli*	Antibacterial	MIC > 64 µg/mL	[29]
	*MRSA*		MIC = 64 µg/mL	
	*P. Aeruginosa*		MIC > 64 µg/mL	
	*V. harveyi*		MIC = 4 µg/mL	
	*V. Parahaemolyticus*		MIC > 64 µg/mL	
	*A. Brassicae*	Antifungal	MIC = 32 µg/mL	
	*C. Cornigerum*		MIC = 64 µg/mL	
	*C. Gloeosporioides*		MIC = 16 µg/mL	
	*C. Gloeosporioides* Penz		MIC = 16 µg/mL	
	*Curvularia spicifera*		MIC = 8 µg/mL	
	*F. Graminearum*		MIC > 64 µg/mL	
	*F. Oxysporum*		MIC = 32 µg/mL	
	*F. Oxysporum* f. Sp. *Radicis lycopersici*		MIC = 32 µg/mL	
	*Fusarium proliferatum*		MIC = 32 µg/mL	
	*P. Digitatum*		MIC = 64 µg/mL	
	*P. Piricola* Nose		MIC = 32 µg/mL	
	*A. hydrophilia*		MIC = 64 µg/mL	
**42**	*B. subtilis*	Antibacterial	MIC > 50 µg/mL	[30]
	*A*. *Baumannii*			
	*E. coil*			
	*MRSA*			
	*C. albicans*	Antifungal	MIC > 50 µg/mL	
**43**	*B. subtilis*	Antibacterial	MIC > 50 µg/mL	[30]
	*A*. *Baumannii*			
	*E. coil*			
	*MRSA*			
	*C. albicans*	Antifungal	MIC > 50 µg/mL	
**44**	*B. subtilis*	Antibacterial	MIC > 50 µg/mL	[30]
	*A*. *Baumannii*			
	*E. coil*			
	*MRSA*			
	*C. albicans*	Antifungal	MIC > 50 µg/mL	
**45**	*B. subtilis*	Antibacterial	MIC 6.25 µg/mL	[30]
	*A*. *Baumannii*		MIC > 50 µg/mL	
	*E. coil*		MIC > 50 µg/mL	
	*MRSA*		MIC > 50 µg/mL	
	*C. albicans*	Antifungal	MIC > 50 µg/mL	
**46**	*B. subtilis*	Antibacterial	MIC > 50 µg/mL	[30]
	*A*. *Baumannii*			
	*E. coil*			
	*MRSA*			
	*C. albicans*	Antifungal	MIC > 50 µg/mL	
**47**	*P. Aeruginosa*	Antibacterial	2.25 ± 0.04 mm AU	[31]
	*E. Faecalis*		1.36 ± 0.04 mm AU	
**48**	*P. Aeruginosa*	Antibacterial	2.8 ± 0.05 mm AU	[31]
	*E. Faecalis*		1.8 ± 0.02 mm AU	

**Table 6:** Antibacterial and/or Antifungal Activities Diterpenoids (**49**–**87**).

No.	Test Strains	Activity	Bioassays	Ref.
**49**	*S. aureus*	Antibacterial	24% (50 µM), 42% (100 µM), 40% (200 µM) inhibition	[33]
**50**	*S. aureus*	Antibacterial	46% (50 µM), 47% (100 µM), 93% (200 µM) inhibition	[33]
**51**	*S. aureus*	Inactive	Inactive against *S. aureus*	[33]
**52**	*V. anguillarum*	Antibacterial	MIC = 12.5 µg/mL	[34]
**53**	*V. anguillarum*	Inactive	Inactive against *V. anguillarum*	[34]
**54**	*V. anguillarum*	Inactive	Inactive against *V. anguillarum*	[34]
**55**	*V. anguillarum*	Inactive	Inactive against *V. anguillarum*	[34]
**56**	*V. anguillarum*	Inactive	Inactive against *V. anguillarum*	[34]
**57**	*V. anguillarum*	Inactive	Inactive against *V. anguillarum*	[34]
**58**	*V. anguillarum*	Inactive	Inactive against *V. anguillarum*	[34]
**59**	*V. anguillarum*	Antibacterial	MIC = 12.5 µg/mL	[34]
**60**	*V. anguillarum*	Inactive	Inactive against *V. anguillarum*	[34]
**61**	*C. albicans*	Antifungal	-	[35]
	*E. coli* ATCC 11775	Inactive	-	
	*S. aureus* ATCC 25923			
	*B. subtilis* ATCC 6633			
**62**	*Exophiala* sp. NJM 1551	Antifungal	MIC = 25 µg/mL	[36]
	*Fusarium moniliforme* NJM 8995		MIC > 100 µg/mL	
	*F. Oxysporum* NJM 0179		MIC = 50 µg/mL	
	*Fusarium solani* NJM 8996		MIC = 50 µg/mL	
	*Haliphthoros sabahensis* IPMB 1402		MIC = 25 µg/mL	
	*H. Milfordensis* IPMB 1603		MIC = 25 µg/mL	
	*Lagenidium thermophilum* IPMB 1401		MIC = 12.5 µg/mL	
**63**	*Exophiala* sp. NJM 1551	Antifungal	MIC = 50 µg/mL	[36]
	*Fusarium moniliforme* NJM 8995		MIC > 100 µg/mL	
	*F. Oxysporum* NJM 0179		MIC > 100 µg/mL	
	*Fusarium solani* NJM 8996		MIC > 100 µg/mL	
	*Haliphthoros sabahensis* IPMB 1402		MIC = 25 µg/mL	
	*H. Milfordensis* IPMB 1603		MIC = 50 µg/mL	
	*Lagenidium thermophilum* IPMB 1401		MIC = 25 µg/mL	
**64**	*Exophiala* sp. NJM 1551	Antifungal	MIC = 50 µg/mL	[36]
	*Fusarium moniliforme* NJM 8995		MIC > 100 µg/mL	
	*F. Oxysporum* NJM 0179		MIC > 100 µg/mL	
	*Fusarium solani* NJM 8996		MIC > 100 µg/mL	
	*Haliphthoros sabahensis* IPMB 1402		MIC = 50 µg/mL	
	*H. Milfordensis* IPMB 1603		MIC = 50 µg/mL	
	*Lagenidium thermophilum* IPMB 1401		MIC = 25 µg/mL	
**65**	*S. aureus*	Antibacterial	0 (12.5 µM), 19.1% (100 µM) inhibition	[37]
**66**	*S. aureus*	Antibacterial	15.3% (12.5 µM), 29.3% (100 µM) inhibition	[37]
**67**	*S. aureus* ATCC 29213	Antibacterial	MIC = 16 µg/mL	[38]
	*S. aureus* USA300LAC		MIC = 16 µg/mL	
	*S. Pneumoniae* ATCC 49619		MIC = 16 µg/mL	
	*S. Pneumoniae* 549 CHUAC		MIC = 32 µg/mL	
	*E. Faecalis* ATCC 29212		MIC = 32 µg/mL	
	*E. Faecalis* 256 CHUAC		MIC > 64 µg/mL	
	*E. Faecium* 214 CHUAC		MIC = 32 µg/mL	
**68**	*S. aureus* ATCC 29213	Antibacterial	MIC = 1 µg/mL	[38]
	*S. aureus* USA300LAC		MIC = 2 µg/mL	
	*S. Pneumoniae* ATCC 49619		MIC = 4 µg/mL	
	*S. Pneumoniae* 549 CHUAC		MIC = 8 µg/mL	
	*E. Faecalis* ATCC 29212		MIC = 4 µg/mL	
	*E. Faecalis* 256 CHUAC		MIC = 4 µg/mL	
	*E. Faecium* 214 CHUAC		MIC = 4 µg/mL	
**69**	*S. aureus* ATCC 29213	Antibacterial	MIC = 8 µg/mL	[38]
	*S. aureus* USA300LAC		MIC = 8 µg/mL	
	*S. Pneumoniae* ATCC 49619		MIC = 16 µg/mL	
	*S. Pneumoniae* 549 CHUAC		MIC > 64 µg/mL	
	*E. Faecalis* ATCC 29212		MIC = 16 µg/mL	
	*E. Faecalis* 256 CHUAC		MIC = 32 µg/mL	
	*E. Faecium* 214 CHUAC		MIC = 8 µg/mL	
**70**	*S. aureus*	Antibacterial	MIC = 8 µg/mL	[39]
	*B. subtilis*		MIC = 4 µg/mL	
**71**	*S. aureus*	Antibacterial	MIC = 8 µg/mL	[39]
	*B. subtilis*		MIC = 4 µg/mL	
**72**	*S. aureus*	Inactive	MIC > 128 µg/mL	[39]
	*B. subtilis*		MIC = 64 µg/mL	
**73**	*S. aureus*	Inactive	MIC > 128 µg/mL	[39]
	*B. subtilis*		MIC = 64 µg/mL	
**74**	*S. aureus*	Inactive	MIC > 128 µg/mL	[39]
	*B. subtilis*			
**75**	*S. aureus*	Inactive	MIC > 128 µg/mL	[39]
	*B. subtilis*		MIC = 64 µg/mL	
**76**	*S. aureus*	Antibacterial	MIC = 32 µg/mL	[40]
	*B. subtilis*		MIC = 32 µg/mL	
	*V. harveyi*		MIC = 64 µg/mL	
	*S. Pneumoniae*		MIC > 128 µg/mL	
	*E. coli*		MIC > 128 µg/mL	
**77**	*S. aureus*	Antibacterial	MIC = 32 µg/mL	[40]
	*B. subtilis*		MIC = 32 µg/mL	
	*V. harveyi*		MIC = 64 µg/mL	
	*S. Pneumoniae*		MIC > 128 µg/mL	
	*E. coli*		MIC > 128 µg/mL	
**78**	*S. aureus*	Antibacterial	MIC = 64 µg/mL	[40]
	*B. subtilis*		MIC = 32 µg/mL	
	*V. harveyi*		MIC > 128 µg/mL	
	*S. Pneumoniae*		MIC > 128 µg/mL	
	*E. coli*		MIC > 128 µg/mL	
**79**	*S. aureus*	Antibacterial	MIC = 64 µg/mL	[40]
	*B. subtilis*		MIC = 64 µg/mL	
	*V. harveyi*		MIC > 128 µg/mL	
	*S. Pneumoniae*		MIC > 128 µg/mL	
	*E. coli*		MIC > 128 µg/mL	
**80**	*S. aureus*	Antibacterial	MIC = 64 µg/mL	[40]
	*B. subtilis*		MIC = 32 µg/mL	
	*V. harveyi*		MIC > 128 µg/mL	
	*S. Pneumoniae*		MIC > 128 µg/mL	
	*E. coli*		MIC > 128 µg/mL	
**81**	*S. aureus*	Antibacterial	MIC = 16 µg/mL	[40]
	*B. subtilis*		MIC = 16 µg/mL	
	*V. harveyi*		MIC > 128 µg/mL	
	*S. Pneumoniae*		MIC > 128 µg/mL	
	*E. coli*		MIC = 8 µg/mL	
**82**	*S. aureus*	Antibacterial	MIC = 32 µg/mL	[40]
	*B. subtilis*		MIC = 16 µg/mL	
	*V. harveyi*		MIC = 64 µg/mL	
	*S. Pneumoniae*		MIC > 128 µg/mL	
	*E. coli*		MIC = 32 µg/mL	
**83**	*S. aureus*	Antibacterial	MIC = 8 µg/mL	[18]
	*B. Cereus*			
**84**	*S. aureus*	Antibacterial	MIC = 4 µg/mL	[18]
	*B. Cereus*		MIC = 16 µg/mL	
**85**	*S. aureus*	Antibacterial	MIC = 4 µg/mL	[18]
	*B. Cereus*		MIC = 16 µg/mL	
**86**	*S. aureus*	Antibacterial	MIC = 16 µg/mL	[18]
	*B. Cereus*		MIC = 8 µg/mL	
**87**	*S. aureus*	Antibacterial	MIC = 16 µg/mL	[18]
	*B. Cereus*		MIC = 8 µg/mL	

“-” indicates that the MIC value is beyond the detectable range.

**Table 7:** Antibacterial and/or Antifungal Activities Triterpenoids (**88**–**105**).

No.	Test Strains	Activity	Bioassays	Ref.
**88**	*S. aureus* USA300LAC	Antibacterial	MIC = 28.1 µg/mL	[41]
	*S. pyogenes* ATCC12344		MIC = 1.8 µg/mL	
**89**	*S. aureus* USA300LAC	Antibacterial	MIC = 30.9 µg/mL	[41]
	*S. pyogenes* ATCC12344		MIC = 1.0 µg/mL	
**90**	*S. aureus* USA300LAC	Antibacterial	MIC > 250.0 µg/mL	[41]
	*S. pyogenes* ATCC12344		MIC = 120.0 µg/mL	
**91**	*S. aureus* USA300LAC	Antibacterial	MIC > 250.0 µg/mL	[41]
	*S. pyogenes* ATCC12344			
**92**	*S. aureus* USA300LAC	Antibacterial	MIC > 250.0 µg/mL	[41]
	*S. pyogenes* ATCC12344		MIC = 65.9 µg/mL	
**93**	*S. aureus* USA300LAC	Antibacterial	MIC > 250.0 µg/mL	[41]
	*S. pyogenes* ATCC12344		MIC = 124.5 µg/mL	
**94**	*S. aureus* USA300LAC	Antibacterial	MIC > 250.0 µg/mL	[41]
	*S. pyogenes* ATCC12344			
**95**	*S. aureus* USA300LAC	Antibacterial	MIC > 250.0 µg/mL	[41]
	*S. pyogenes* ATCC12344			
**96**	*S. aureus* USA300LAC	Antibacterial	MIC > 250.0 µg/mL	[41]
	*S. pyogenes* ATCC12344		MIC = 240.0 µg/mL	
**98**	*S. aureus*	Antibacterial	MIC > 100 µg/mL	[42]
**100**	*S. aureus*	Antibacterial	MIC > 100 µg/mL	[42]
**101**	*S. aureus*	Antibacterial	MIC > 100 µg/mL	[42]
**102**	*S. aureus*	Antibacterial	MIC > 100 µg/mL	[42]
**104**	*S. aureus*	Antibacterial	MIC = 2.5 µg/mL	[42]
**105**	*S. aureus*	Antibacterial	MIC = 0.078 µg/mL	[42]

**Table 8:** Antibacterial and/or antifungal activities meroterpenoids (**108**–**141**).

No.	Test Strains	Activity	Bioassays	Ref.
**108**	*A. hydrophilia*	Antibacterial	MIC = 32 µg/mL	[46]
	*E. coli*		MIC = 16 µg/mL	
	*E. Tarda*		MIC = 0.5 µg/mL	
	*V. anguillarum*		MIC = 0.5 µg/mL	
	*V. harveyi*		MIC = 32 µg/mL	
	*C. Diplodiella*,	Antifungal	MIC = 8 µg/mL	
	*F. Graminearum*		MIC = 32 µg/mL	
**109**	*A. hydrophilia*	Antibacterial	MIC = 16 µg/mL	
	*E. coli*		MIC = 1 µg/mL	[46]
	*E. Tarda*		MIC = 32 µg/mL	
	*V. anguillarum*		MIC = 4 µg/mL	
	*V. harveyi*		MIC = 32 µg/mL	
	*C. Diplodiella*	Antifungal	MIC = 64 µg/mL	
	*F. Graminearum*			
**110**	*A. hydrophilia*	Antibacterial	MIC = 32 µg/mL	[46]
	*E. coli*		MIC = 32 µg/mL	
	*E. Tarda*		MIC = 16 µg/mL	
	*V. anguillarum*		MIC = 32 µg/mL	
	*V. harveyi*		MIC = 16 µg/mL	
	*C. Diplodiella*	Antifungal	MIC > 64 µg/mL	
	*F. Graminearum*		MIC > 32 µg/mL	
**111**	*A. hydrophilia*	Antibacterial	MIC = 32 µg/mL	[46]
	*E. coli*		MIC = 16 µg/mL	
	*E. Tarda*		MIC = 0.5 µg/mL	
	*V. anguillarum*		MIC = 0.5 µg/mL	
	*V. harveyi*		MIC = 32 µg/mL	
	*C. Diplodiella*	Antifungal	MIC = 8 µg/mL	
	*F. Graminearum*		MIC = 32 µg/mL	
**112**	*S. aureus* ATCC6538P	Antibacterial	MIC = 15.2 µg/mL	[47]
	*E. coli*		-	
	*V. Parahaemolyticus*	Antifungal	-	
**116**	*S. aureus* CICC 10384	Antibacterial	MIC = 12.5 µg/mL	[48]
**119**	*M. lutea*	Antibacterial	MIC = 6.25 µg/mL	[49]
	*K. Pneumoniae*		MIC = 50 µg/mL	
	*MRSA*		MIC = 6.25 µg/mL	
	*S. faecalis*		MIC = 6.25 µg/mL	
**120**	*M. lutea*	Antibacterial	MIC = 25 µg/mL	[49]
	*K. Pneumoniae*		MIC > 100 µg/mL	
	*MRSA*		MIC = 6.25 µg/mL	
	*S. faecalis*		MIC = 25 µg/mL	
**121**	*M. lutea*	Antibacterial	MIC = 25 µg/mL	[49]
	*K. Pneumoniae*		MIC = 25 µg/mL	
	*MRSA*		MIC = 12.5 µg/mL	
	*S. faecalis*		MIC > 100 µg/mL	
**122**	*M. lutea*	Antibacterial	MIC > 100 µg/mL	[49]
	*K. Pneumoniae*		MIC = 6.25 µg/mL	
	*MRSA*		MIC = 25 µg/mL	
	*S. faecalis*		MIC = 50 µg/mL	
**123**	*M. lutea*	Antibacterial	MIC = 12.5 µg/mL	[49]
	*K. Pneumoniae*		MIC > 100 µg/mL	
	*MRSA*		MIC = 12.5 µg/mL	
	*S. faecalis*		MIC = 12.5 µg/mL	
**124**	*M. lutea*	Inactive	MIC > 100 µg/mL	[49]
	*K. Pneumoniae*			
	*MRSA*			
	*S. faecalis*			
**125**	*S. aureus* ATCC6538	Antibacterial	MIC > 128 µg/mL	[50]
	*B. subtilis* ATCC 6633			
	*E. coli* ATCC 25922			
	*L. Monocytogenes* ATCC 1911			
	*F. Oxysporum* f. Sp. Cubense	Inactive	MIC > 128 µg/mL	
	*Fusarium* spp.			
	*P. Litchii*			
	*C. Gloeosporioides*			
	*H. Undatus*			
**126**	*S. aureus* ATCC6538	Antibacterial	MIC > 128 µg/mL	[50]
	*B. subtilis* ATCC 6633			
	*E. coli* ATCC 25922			
	*L. Monocytogenes* ATCC 1911			
	*F. Oxysporum* f. Sp. Cubense	Inactive	MIC > 128 µg/mL	
	*Fusarium* spp.			
	*P. Litchii*			
	*C. Gloeosporioides*			
	*H. Undatus*			
**127**	*S. aureus* ATCC6538	Antibacterial	MIC > 128 µg/mL	[50]
	*B. subtilis* ATCC 6633			
	*E. coli* ATCC 25922			
	*L. Monocytogenes* ATCC 1911			
	*F. Oxysporum* f. Sp. Cubense	Inactive	MIC > 128 µg/mL	
	*Fusarium* spp.			
	*P. Litchii*			
	*C. Gloeosporioides*			
	*H. Undatus*			
**128**	*S. aureus* ATCC6538	Antibacterial	MIC > 128 µg/mL	[50]
	*B. subtilis* ATCC 6633			
	*E. coli* ATCC 25922			
	*L. Monocytogenes* ATCC 1911			
	*F. Oxysporum* f. Sp. Cubense	Inactive	MIC > 128 µg/mL	
	*Fusarium* spp.			
	*P. Litchii*			
	*C. Gloeosporioides*			
	*H. Undatus*			
**129**	*S. aureus* ATCC6538	Antibacterial	MIC = 128 µg/mL	[50]
	*B. subtilis* ATCC 6633		MIC > 128 µg/mL	
	*E. coli* ATCC 25922		MIC > 128 µg/mL	
	*L. Monocytogenes* ATCC 1911		MIC > 128 µg/mL	
	*F. Oxysporum* f. Sp. Cubense	Inactive	Inactive against *F. Oxysporum* f. Sp. Cubense	
	*Fusarium* spp.		Inactive against *Fusarium* spp.	
	*P. Litchii*		Inactive against *P. Litchii*	
	*C. Gloeosporioides*		Inactive against *C. Gloeosporioides*	
	*H. Undatus*		Inactive against *H. Undatus*	
**130**	*S. aureus* ATCC6538	Antibacterial	MIC > 128 µg/mL	[50]
	*B. subtilis* ATCC 6633			
	*E. coli* ATCC 25922			
	*L. Monocytogenes* ATCC 1911			
	*F. Oxysporum* f. Sp. Cubense	Inactive	MIC > 128 µg/mL	
	*Fusarium* spp.			
	*P. Litchii*			
	*C. Gloeosporioides*			
	*H. Undatus*			
**131**	*S. aureus* ATCC6538	Antibacterial	MIC > 128 µg/mL	[50]
	*B. subtilis* ATCC 6633			
	*E. coli* ATCC 25922			
	*L. Monocytogenes* ATCC 1911			
	*F. Oxysporum* f. Sp. Cubense	Inactive	MIC > 128 µg/mL	
	*Fusarium* spp.			
	*P. Litchii*			
	*C. Gloeosporioides*			
	*H. Undatus*			
**132**	*S. aureus* ATCC6538	Antibacterial	MIC > 128 µg/mL	[50]
	*B. subtilis* ATCC 6633			
	*E. coli* ATCC 25922			
	*L. Monocytogenes* ATCC 1911			
	*F. Oxysporum* f. Sp. Cubense	Inactive	MIC > 128 µg/mL	
	*Fusarium* spp.			
	*P. Litchii*			
	*C. Gloeosporioides*			
	*H. Undatus*			
**133**	*MRSA*	Antibacterial	MIC = 25 µg/mL	[20]
	*B. subtilis*		MIC = 6.25 µg/mL	
	*P. Aeruginosa*		MIC > 50 µg/mL	
	*S. Typhimurium*		MIC > 50 µg/mL	
	*P. Italicum*	Antifungal	MIC = 6.25 µg/mL	
	*C. Gloeosporioides*			
**134**	*MRSA*	Antibacterial	MIC = 25 µg/mL	[20]
	*B. subtilis*		MIC = 25 µg/mL	
	*P. Aeruginosa*		MIC = 25 µg/mL	
	*S. Typhimurium*		MIC > 50 µg/mL	
	*P. Italicum*	Antifungal	MIC = 50 µg/mL	
	*C. Gloeosporioides*		MIC > 50 µg/mL	
**135**	*MRSA*	Antibacterial	MIC > 50 µg/mL	[20]
	*B. subtilis*			
	*P. Aeruginosa*			
	*S. Typhimurium*			
	*P. Italicum*	Antifungal	MIC = 50 µg/mL	
	*C. Gloeosporioides*		MIC > 50 µg/mL	
**136**	*B. subtilis* KCTC 1021	Antibacterial	MIC = 16 µg/mL	[51]
	*K. Rhizophila* KCTC 1915		MIC = 32 µg/mL	
	*S. aureus* KCTC 1927		MIC = 16 µg/mL	
	*E. coli* KCTC 2441		MIC > 128 µg/mL	
	*S. Typhimurium* KCTC 2515		MIC > 128 µg/mL	
	*K. Pneumonia* KCTC 2690		MIC > 128 µg/mL	
**137**	*B. subtilis* KCTC 1021	Antibacterial	MIC = 64 µg/mL	[51]
	*K. Rhizophila* KCTC 1915		MIC > 128 µg/mL	
	*S. aureus* KCTC 1927		MIC > 128 µg/mL	
	*E. coli* KCTC 2441		MIC > 128 µg/mL	
	*S. Typhimurium* KCTC 2515		MIC > 128 µg/mL	
	*K. Pneumonia* KCTC 2690		MIC > 128 µg/mL	
**138**	*B. subtilis* KCTC 1021	Antibacterial	MIC = 1 µg/mL	[51]
	*K. Rhizophila* KCTC 1915		MIC = 2 µg/mL	
	*S. aureus* KCTC 1927		MIC = 2 µg/mL	
	*E. coli* KCTC 2441		MIC > 128 µg/mL	
	*S. Typhimurium* KCTC 2515		MIC > 128 µg/mL	
	*K. Pneumonia* KCTC 2690		MIC > 128 µg/mL	
**139**	*B. subtilis* KCTC 1021	Antibacterial	MIC > 128 µg/mL	[51]
	*K. Rhizophila* KCTC 1915			
	*S. aureus* KCTC 1927			
	*E. coli* KCTC 2441			
	*S. Typhimurium* KCTC 2515			
	*K. Pneumonia* KCTC 2690			
**140**	*H. pylori* ATCC43504	Antibacterial	MIC = 2 µg/mL	[52]
	*H. pylori* G27		MIC = 1 µg/mL	
	*H. pylori* Hp159		MIC = 1 µg/mL	
	*H. pylori* BY583		MIC = 4 µg/mL	
	*S. aureus* ATCC25923		MIC = 16 µg/mL	
	*S. aureus* USA300		MIC = 2 µg/mL	
	*S. aureus* BKS231		MIC = 4 µg/mL	
	*S. aureus* BKS233		MIC = 8 µg/mL	
**141**	*E. coli*	Antibacterial	MIC = 8 µg/mL	[53]
	*M. Luteus*		MIC = 8 µg/mL	
	*P. Aeruginosa*		MIC = 16 µg/mL	
	*R. Solanacearum*		MIC = 8 µg/mL	
	*A. Alternata*	Antifungal	MIC > 64 µg/mL	
	*B. Cinerea*		MIC = 32 µg/mL	
	*F. Oxysporum*		MIC > 64 µg/mL	
	*P. Digitatum*		MIC = 32 µg/mL	
	*V. Mali*		MIC = 16 µg/mL	

“-” indicates that the MIC value is beyond the detectable range.

## Data Availability

Not applicable.

## Abbreviations

SAR	structure–activity relationships
MIC	minimum inhibitory concentration
*VRE*	vancomycin-resistant *Enterococcus faecalis*
*MRSA*	methicillin-resistant *Staphylococcus aureus*
*K. pneumoniae*	*Klebsiella pneumoniae*
*A. baumannii*	*Acinetobacter baumannii*
*P. aeruginosa*	*Pseudomonas aeruginosa*
*E. coli*	*Escherichia coli*
*PRSP*	penicillin-resistant *Streptococcus pneumoniae*
XDR	extensively drug-resistant
NMR	nuclear magnetic resonance
MS	mass spectroscopic
QM	quantum mechanics
TDDFT	time-dependent density functional theory
ECD	electronic circular dichroism
HRESIMS	high-resolution electrospray ionization mass spectrometry
CD	circular dichroism
VCD	vibrational circular dichroism
NOESY	nuclear Overhauser effect spectroscopy
HSQC	heteronuclear single quantum coherence
HMBC	heteronuclear multiple bond correlation
NOE	nuclear Overhauser effect
*M. tuberculosis*	*Mycobacterium tuberculosis*
*V. harveyi*	*Vibrio harveyi*
*A. brassicae*	*Alternaria brassicae*
*C. cornigerum*	*Calonectria cornigerum*
*C. gloeosporioides*	*Colletotrichum gloeosporioides*
*B. cereus*	*Bacillus cereus*
*S. aureus*	*Staphylococcus aureus*
*A. niger*	*Aspergillus niger*
*F. oxysporum*	*Fusarium oxysporum*
*C. albicans*	*Candida albicans*
*C. difficile*	*Clostridium difficile*
*P. italicum*	*Penicillium italicum*
*V. parahaemolyticus*	*Vibrio parahaemolyticus*
*B. megaterium*	*Bacillus megaterium*
*M. luteus*	*Micrococcus luteus*
*S. pyogenes*	*Streptococcus pyogenes*
*E. faecium*	*Enterococcus faecium*
*B. subtilis*	*Bacillus subtilis*
*S. typhimurium*	*Salmonella typhimurium*
*E. faecalis*	*Enterococcus faecalis*
*V. anguillarum*	*Vibrio* *anguillarum*
IC_50_	half-maximal inhibitory concentration
*S. pneumoniae*	*Streptococcus pneumoniae*
*A. hydrophilia*	*Aeromonas hydrophilia*
*E. tarda*	*Edwardsiella tarda*
*C. diplodiella*	*Coniothyrium diplodiella*
*F. graminearum*	*Fusarium graminearum*
EtOAc	ethyl acetate
*L. monocytogenes*	*Listeria monocytogenes*
*P. litchii*	*Peronophythora litchii*
*S. faecalis*	*Streptococcus faecalis*
*H. undatus*	*Hylocereus undatus*
*H. pylori*	*Helicobacter pylori*
*R. solanacearum*	*Ralstonia solanacearum*
*A. alternata*	*Alternaria alternata*
*B. cinerea*	*Botrytis cinerea*
*P. digitatum*	*Penicillium digitatum*
*V. mali*	*Valsa mali*
